# A structural accessibility principle for LbuCas13a activation by noncontiguous DNA

**DOI:** 10.1093/nar/gkag740

**Published:** 2026-07-25

**Authors:** Weitao Wang, Yuhan Chen, Ziyun Li, Li Zhang, Kai Gui, You Wu, Na Yin, Xiaole Han, Yaoyi Zhang, Ruiling Lu, Ziheng Zhang, Li Wang, Guoming Xie

**Affiliations:** Department of Neurosurgery, The First Affiliated Hospital of Chongqing Medical University, No.1 Youyi Road, Chongqing 400016, P.R. China; Key Laboratory of Clinical Laboratory Diagnostics (Chinese Ministry of Education), College of Laboratory Medicine, Chongqing Medical University, Chongqing 400016, P.R. China; Key Laboratory of Clinical Laboratory Diagnostics (Chinese Ministry of Education), College of Laboratory Medicine, Chongqing Medical University, Chongqing 400016, P.R. China; Key Laboratory of Clinical Laboratory Diagnostics (Chinese Ministry of Education), College of Laboratory Medicine, Chongqing Medical University, Chongqing 400016, P.R. China; Key Laboratory of Clinical Laboratory Diagnostics (Chinese Ministry of Education), College of Laboratory Medicine, Chongqing Medical University, Chongqing 400016, P.R. China; Key Laboratory of Clinical Laboratory Diagnostics (Chinese Ministry of Education), College of Laboratory Medicine, Chongqing Medical University, Chongqing 400016, P.R. China; Key Laboratory of Clinical Laboratory Diagnostics (Chinese Ministry of Education), College of Laboratory Medicine, Chongqing Medical University, Chongqing 400016, P.R. China; Key Laboratory of Clinical Laboratory Diagnostics (Chinese Ministry of Education), College of Laboratory Medicine, Chongqing Medical University, Chongqing 400016, P.R. China; Key Laboratory of Clinical Laboratory Diagnostics (Chinese Ministry of Education), College of Laboratory Medicine, Chongqing Medical University, Chongqing 400016, P.R. China; Key Laboratory of Clinical Laboratory Diagnostics (Chinese Ministry of Education), College of Laboratory Medicine, Chongqing Medical University, Chongqing 400016, P.R. China; Key Laboratory of Clinical Laboratory Diagnostics (Chinese Ministry of Education), College of Laboratory Medicine, Chongqing Medical University, Chongqing 400016, P.R. China; Key Laboratory of Clinical Laboratory Diagnostics (Chinese Ministry of Education), College of Laboratory Medicine, Chongqing Medical University, Chongqing 400016, P.R. China; The Center for Clinical Molecular Medical Detection, Engineering Research Center of Chongqing Education Commission of China for IVD Technology Innovation and Translation, Laboratory Medicine Center, The First Affiliated Hospital of Chongqing Medical University, Chongqing 400016, P.R. China; Department of Neurosurgery, The First Affiliated Hospital of Chongqing Medical University, No.1 Youyi Road, Chongqing 400016, P.R. China; Key Laboratory of Clinical Laboratory Diagnostics (Chinese Ministry of Education), College of Laboratory Medicine, Chongqing Medical University, Chongqing 400016, P.R. China

## Abstract

CRISPR–Cas13a is mainly known as an RNA-guided RNA endonuclease. Recent studies show that *Leptotrichia buccalis* Cas13a (LbuCas13a) can interact with DNA substrates too, without PAM or PFS constraints, but current understanding of DNA-mediated activation is largely based on continuous target strands. Here, we define a structural accessibility principle for LbuCas13a activation by noncontiguous DNA. We show that activation occurs only when overhang positioning creates an accessible protein–DNA interface. Outer overhangs near the crRNA repeat-adjacent side restore strong *trans*-cleavage activity by stabilizing key LbuCas13a–DNA contacts, whereas distal outer overhangs support only weak activation. In contrast, inner overhangs cause steric mismatch, destabilize the complex, and block formation of an active conformation. Molecular modeling and molecular dynamics simulations support this structure-dependent rule. Noncontiguous DNA also broadens the single-nucleotide discrimination window of LbuCas13a and enables accurate IDH1 R132H detection in glioma tissues. We further develop a one-step APE1-activated CRISPR–LbuCas13a reaction (ACROSS) for sensitive APE1 detection. Because activated LbuCas13a cleaves RNA reporters but not DNA-triggering products, ACROSS preserves the activating structure and supports stable signaling *in vitro*, in live cells, and in breast cancer serum samples.

## Graphical Abstract

**Graphical Abstract:**
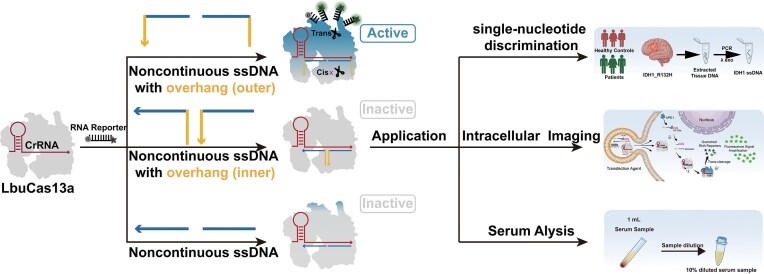

## Introduction

CRISPR–Cas13a is a class 2, Type VI-A effector [1–3] characterized by RNA-induced target recognition and secondary cleavage activity, enabling numerous applications in nucleic acid detection and molecular imaging [4–9]. Cas13a activity is strictly dependent on target-induced activation, and the HEPN catalytic domain must adopt an activatable conformation to initiate cleavage [10, 11]. Recent studies have demonstrated that Cas13a from *Leptotrichia buccalis* (LbuCas13a) is directly activated by DNA substrates without the constraints of protospacer adjacent motif (PAM) or protospacer flanking site (PFS) [12, 13]. This finding expands Cas13a’s molecular recognition capabilities beyond RNA, opening new avenues for the detection of non-RNA targets.

Current activation models assume that target sequences are continuous, and this is often used as a basic design assumption [14–17]. But in biological and analytical settings, nucleic acids are rarely present as perfect continuous strands [18, 19]. In real samples, accessible sequence regions may be separated in space or restricted by structure. This can reduce their interaction with the Cas13a–substrate complex [20, 21]. These scenarios challenge the implicit assumption that Cas13a activation requires a continuous target structure. Therefore, the question remains whether the failure of activation in noncontinuous structures is due to a lack of sequence information or structural constraints imposed on the Cas13a–substrate complex.

In this study, we systematically investigated the structural requirements necessary for LbuCas13a activation using cleaved single-stranded DNA as a model system [22–24]. The results showed that LbuCas13a activation by noncontinuous DNA follows a structural accessibility principle: noncontinuous DNA is not activated solely by restoring local sequence continuity or by the presence of an overhang, but requires a configuration that provides a structurally accessible interface for productive LbuCas13a engagement. Accordingly, activation was restored only when an outer overhang was positioned in a manner that preserved this structural accessibility. Outer overhangs proximal to the crRNA repeat-adjacent region effectively restored *trans*-cleavage activity, whereas distal outer overhangs supported only weak or unstable activation. In contrast, inner overhangs markedly impaired activation, consistent with their structural incompatibility with the formation of a stable, activation-competent LbuCas13a–DNA complex.

Molecular dynamics (MD) simulations revealed that outer overhangs located near the interface adjacent to the crRNA repeat play a crucial role in stabilizing key interaction surfaces and maintaining a complex structure suitable for activation. In contrast, distal outer overhangs have little effect on interface stabilization, while inner overhangs introduce steric hindrance to critical interaction regions, destabilizing the complex and inhibiting efficient activation. Collectively, these findings establish a structural accessibility rule governing noncontiguous DNA-mediated activation of LbuCas13a. Based on this structure-guided activation principle, we further explored its utility for single-nucleotide variant discrimination and developed the APE1 (apurinic/apyrimidinic endonuclease 1)-activated CRISPR–LbuCas13a Reaction in One-Step System (ACROSS), and applied it to the detection of APE1. ACROSS enabled sensitive *in vitro* detection and was further adapted for intracellular imaging and serum analysis from breast cancer (BC) patients. Notably, because activated LbuCas13a does not cleave DNA products, the triggering structure within ACROSS remains intact, preventing signal self-consumption and ensuring stable signal output in complex biological environments.

## Materials and methods

### Ethical statement

All subjects gave their informed consent for inclusion before they participated in the study. The study was conducted in accordance with the Declaration of Helsinki, and the protocol was approved by the Ethics Committee of Chongqing Medical University (No. 2023-280).

### Materials and reagents

LbuCas13a protein was purchased from Bio-Lifesci. All DNA and RNA oligonucleotides (Supplementary Table S1) were synthesized with HPLC (high performance liquid chromatography ) purification by Sangon Biotech (Shanghai, China). Apurinic/apyrimidinic Endonuclease 1 (APE1), NEB 4 reaction buffer, uracil-DNA glycosylase (UDG), flap endonuclease 1 (FEN1), λ exonuclease, and Nt.BbvCI were obtained from New England Biolabs (Ipswich, MA, USA). Lipofectamine 3000 transfection reagent, N,N,N′,N′-tetramethylethylenediamine (TEMED), and 30% acrylamide/bis-acrylamide solution were purchased from Thermo Fisher Scientific (Waltham, MA, USA). The 25–500 bp DNA marker, GelRed nucleic acid stain, and 6× loading buffer were obtained from Sangon Biotech (Shanghai, China). The human apurinic/apyrimidinic endonuclease 1 (APE1) ELISA kit was purchased from ELK Biotechnology (Wuhan, China). High-glucose Dulbecco’s modified Eagle’s medium (DMEM) was purchased from Gibco (Thermo Fisher Scientific, Waltham, MA, USA). Fetal bovine serum (FBS) was obtained from Lonsera (Montevideo, Uruguay), and penicillin–streptomycin solution (BL505A) was purchased from Biosharp (Hefei, China). Human embryonic kidney cells (HEK293T), human breast cancer cells (MCF-7), human hepatocellular carcinoma cells (Huh-7), and human cervical cancer cells (HeLa) were obtained from the American Type Culture Collection (ATCC, Manassas, VA, USA). All cell lines were maintained by the Key Laboratory of Clinical Laboratory Diagnostics, Ministry of Education, College of Laboratory Medicine, Chongqing Medical University, and cultured in a humidified incubator at 37°C with 5% CO₂.

Oligonucleotides were annealed using a thermal cycler (CFX96, Bio-Rad, USA). Samples were heated at 95°C for 5 min and cooled from 95°C to 12°C at a rate of 0.1°C s⁻¹. All experiments were performed with three independent replicates per condition. Real-time fluorescence measurements were conducted using a Rotor-Gene 6000 instrument (Corbett Research, Mortlake, Australia) at 37°C with default gain settings, and fluorescence signals were collected in the yellow channel. Gel images were acquired using a ChemiDoc XRS imaging system (Bio-Rad Laboratories, USA) and analyzed with Image Lab software (Bio-Rad). Confocal fluorescence images were obtained using a laser scanning confocal microscope (Leica TCS SP8, Leica Microsystems). Absorbance at 450 nm (OD_450_) was measured using a microplate reader (BioTek, USA).

### Design and *in silico* prediction of nucleic acid architectures

Secondary structures and thermodynamic properties of the designed DNA and RNA strands were analyzed using NUPACK (v4.0). Calculations were performed based on the nucleic acid secondary structure statistical thermodynamic model, incorporating strand–strand interactions at specified concentrations. Unless otherwise stated, the temperature was set to 37°C. Sequence design quality was evaluated by calculating the minimum free energy structures, equilibrium base-pairing probabilities, and ensemble defect of the target complexes.

### 12% native polyacrylamide gel electrophoresis

Twenty-five milliliters of 12% native polyacrylamide gels were prepared before. Five microliter sample solution was mixed with 1 μl of 6× loading buffer and then loaded onto the 12% native polyacrylamide gel electrophoresis (PAGE). Electrophoresis was performed in 1× TBE buffer [2 mM ethylenediaminetetraacetic acid (EDTA) and 89 mM Tris–boric acid, pH 8.3] at a 100-V constant voltage for 60 min before staining with 4S GelRed. Finally, the gel was visualized under UV light by Bio-Rad ChemiDoc XRS imaging system (Bio-Rad Laboratories, American).

### Preparation for CRISPR–LbuCas13a reaction

An optimized 20 μl CRISPR–LbuCas13a reaction mixture was prepared containing 10 nM Cas13a, 10 nM crRNA, 250 nM RNA reporter, and 1× reaction buffer. A total of 18 μl of the reaction mixture was dispensed into the bottom of each tube, followed by the addition of 2 μl of target (DNA or RNA) onto the inner wall of the tube. After brief centrifugation to initiate the reaction, the tubes were immediately placed in Rotor-Gene 6000 instrument for real-time fluorescence measurement at 37°C. Fluorescence signals were recorded every 20 s for 60 min.

### Molecular dynamics simulations and binding free energy analysis

#### Initial complex construction

Protein-DNA complexes were generated using the amino acid sequence of Cas13a and eight DNA probe variants (S1-S8). AlphaFold3 was employed to predict the structures of the protein–DNA complexes by co-submitting the Cas13a sequence together with each corresponding DNA sequence. The top-ranked model for each complex was selected as the initial structure for subsequent MD simulations.

#### System preparation and simulation protocol

All complexes were prepared using the CHARMM-GUI Solution Builder. Each system was solvated using the TIP3P water model and supplemented with 150 mM NaCl to mimic physiological ionic conditions. MD simulations were performed using GROMACS (v2022). The AMBER ff19SB force field was applied for the protein, and the OL15 force field was used for DNA. Each system underwent energy minimization followed by sequential equilibration under the NVT and NPT ensembles with gradually released positional restraints. Production simulations were then carried out for 100 ns under conditions of 298.15 K and 1 bar without restraints.

#### Trajectory analysis

Trajectory analyses were conducted using GROMACS utilities. Structural stability and flexibility were evaluated by calculating the Cα root-mean-square deviation (RMSD) and root-mean-square fluctuation (RMSF). The radius of gyration (Rg) and solvent-accessible surface area (SASA) were analyzed to assess global conformational changes. Hydrogen bonds between Cas13a and DNA were quantified to evaluate intermolecular interactions.

#### MM/GBSA binding free energy calculation

Binding free energies (ΔG_bind) of the Cas13a–DNA complexes were estimated using the MM/GBSA method. After system equilibration, 2000 frames were uniformly extracted from each trajectory for free energy calculation. Per-residue energy decomposition analysis was performed to identify key residues contributing to protein–DNA interactions.

### Calculation of discrimination factor

The discrimination factor (DF) was calculated using the following equation: DF = (Fwt − Fp)/(Fmt − Fp), where Fwt, Fmt, and Fp denote the fluorescence signals generated by the perfectly matched target, the mutant target, and the probe-only control, respectively.

### Fluorescence assay for the ACROSS system

The P-AP-a14-o8 and P1-8 strands were dissolved in TEMg buffer (10 mM Tris–HCl, 1 mM EDTA, 10 mM MgCl₂, pH 7.5) to final concentrations of 2 and 2.5 μM, respectively. The mixture was annealed in a thermal cycler by heating at 95°C for 5 min, followed by slow cooling to 25°C at a rate of 0.1°C s⁻¹ and incubation at 25°C for 10 min to form a stable duplex structure. The annealed product was stored at 4°C until use.

For ACROSS detection, all reaction components were combined simultaneously in a single 20-μl reaction mixture prepared in 200 μl PCR (polymerase chain reaction) tubes. The reaction contained NEBuffer 4 (1×), annealed APS substrate (100 nM), APE1 enzyme (0.1 U μl⁻¹), LbuCas13a (50 nM), crRNA (50 nM), b14-o8 strand (10 nM), RNA reporter (500 nM), and nuclease-free water to a final volume of 20 μl. After gentle mixing and brief centrifugation, the reaction mixture was immediately transferred to a Rotor-Gene 6000 instrument for real-time fluorescence measurement at 37°C.

### Bioinformatic analysis of APE1 expression

APE1 (encoded by APEX1) mRNA expression data from tumor tissues were obtained from The Cancer Genome Atlas (TCGA, https://www.cancer.gov/ccg/research/genome-sequencing/tcga) RNA-seq datasets, whereas normal tissue data were obtained from the Genotype-Tissue Expression (GTEx, https://www.gtexportal.org/home/) project to compensate for the limited number of normal samples in TCGA. The TCGA and GTEx datasets were accessed through the UCSC Xena platform (https://xena.ucsc.edu/) and the GTEx Portal, respectively. APE1 protein expression data for the corresponding cancer types were obtained from the Clinical Proteomic Tumor Analysis Consortium (CPTAC, https://gdc.cancer.gov/) public proteomics datasets through the CPTAC Data Portal. Before analysis, mRNA expression data were log_2_-transformed, whereas protein expression data were normalized using *z*-scores. APE1 expression distributions were presented as box plots, in which the center line indicates the median, the box boundaries indicate the first and third quartiles, and individual dots represent individual samples. Differences between tumor and normal tissues were assessed using a two-sided Wilcoxon rank-sum test. *P* < .05 was considered statistically significant.

### Cell culture and intracellular imaging experiments

#### Cell culture and protein extraction

HEK293T, MCF-7, Huh-7, and HeLa cells were cultured at 37°C in a humidified incubator with 5% CO₂ in high-glucose DMEM supplemented with 10% FBS and 1% penicillin–streptomycin. At 80%–90% confluence, cells were detached using trypsin and washed twice with phosphate-buffered saline (PBS). For cell lysate preparation, harvested cells were resuspended in RIPA lysis buffer supplemented with protease inhibitor cocktail and incubated on ice for 30 min with vortexing every 10 min. The lysates were centrifuged at 12 000 × *g* for 20 min at 4°C, and the supernatants were collected as total protein extracts. Protein concentrations were determined using a BCA protein assay kit. Aliquots were stored at −80°C until use.

#### Fluorescence kinetic analysis across varying cell numbers and cell types

To evaluate the response of the ACROSS system to cell-derived APE1, a 20-μl reaction mixture was prepared in 200-μl PCR tubes containing LbuCas13a (50 nM), crRNA (50 nM), APS (AP-Switch, 100 nM), b14-o8 strand (10 nM), varying amounts of cell lysate or equal total protein amounts from different cell lines (MCF-7, Huh-7, HeLa), Cas13a reaction buffer (1×), and FQ-reporter (500 nM). The volume was adjusted to 20 μl with nuclease-free water. Following brief centrifugation, the reactions were immediately transferred to Rotor-Gene 6000 instrument for real-time fluorescence measurement at 37°C. Fluorescence signals were recorded every 20 s for 30 min.

#### Intracellular imaging of ACROSS activity

For intracellular imaging, Huh-7, MCF-7, HeLa, or HEK293T cells were seeded in confocal dishes at a density of 1 × 10⁵ cells per well 24 h prior to transfection and cultured in DMEM containing 10% FBS. At 70%–80% confluence, cells were co-transfected using Lipofectamine 3000 according to the manufacturer’s instructions. The transfection mixtures contained LbuCas13a ribonucleoprotein complex (RNP) (100 nM), APS (100 nM), b14-o8 strand (50 nM), and FQ-reporter (500 nM), combined according to the experimental groups. After 4 h, the medium was replaced with fresh culture medium, and cells were further incubated for 24 h. To assess intracellular ACROSS activity, Huh-7 cells were assigned to four groups: (I) RNP + FQ-reporter; (II) RNP + b14-o8 + FQ-reporter; (III) RNP + APS + FQ-reporter; and (IV) RNP + APS + b14-o8 + FQ-reporter. To evaluate applicability across different tumor cell types, group IV was transfected into MCF-7, Huh-7, HeLa, and HEK293T cells. Twenty-four hours post-transfection, cells were washed three times with PBS and imaged using a laser scanning confocal microscope. At least five random fields were acquired per sample. Fluorescence intensities were quantified using ImageJ, and mean fluorescence intensity was calculated.

### Clinical serum sample collection and preparation

A total of 77 serum samples were collected in this study, including 47 serum samples from pathologically confirmed BC patients (breast cancer group, BC) and 30 serum samples from healthy individuals undergoing routine physical examination (healthy donor group, HD). All samples were collected at the First Affiliated Hospital of Chongqing Medical University between October 1, 2025 and February 1, 2026. Written informed consent was obtained from all participants. The study was approved by the Ethics Committee of Chongqing Medical University (approval number: No. 2023-280). Peripheral venous blood samples were collected in vacuum tubes without anticoagulant and allowed to clot at room temperature for 30 min. Samples were then centrifuged at 3000 rpm for 15 min at 4°C. The supernatant serum was carefully collected and aliquoted into sterile centrifuge tubes to avoid repeated freeze–thaw cycles. All serum samples were stored at −80°C until use. Before analysis, frozen serum samples were thawed at 4°C, vortexed gently, and briefly centrifuged. To minimize matrix interference, all serum samples were diluted tenfold in sterile 1× PBS buffer (pH 7.4) to obtain a 10% serum working solution (serum:PBS = 1:9, total volume 100 μl). Diluted samples were kept on ice and analyzed within 2 h.

### Analysis of clinical samples by ACROSS

To evaluate the performance of the ACROSS system in clinical samples, APE1 levels in the 77 collected serum samples (47 BC and 30 HD) were analyzed using the ACROSS platform. The ACROSS reaction mixture (total volume 20 μl) was prepared in 200 μl PCR tubes containing LbuCas13a (50 nM), crRNA (50 nM), APS (100 nM), b14-o8 strand (10 nM), FQ-reporter (500 nM), and Cas13a reaction buffer (1×). Prior to detection, all serum samples were diluted to 10% in sterile 1× PBS. Two microliters of diluted serum was added to the reaction mixture, and the final volume was adjusted to 20 μl with nuclease-free water. Each sample was tested in triplicate. A negative control was prepared by substituting PBS for serum. Reactions were incubated at 37°C for 1 h, and fluorescence kinetics were recorded every 20 s using a fluorescence microplate reader. For comparison, the same batch of serum samples was analyzed using a commercial human APE1 ELISA kit according to the manufacturer’s instructions. OD_450_ was measured for quantitative comparison.

### ELISA analysis

A commercial human APE1 ELISA kit was used according to the manufacturer’s instructions. All reagents were equilibrated to room temperature prior to use. Wash buffer, standard dilution buffer, and substrate working solution were prepared as instructed. Lyophilized standards were reconstituted in standard dilution buffer and serially diluted to generate a standard curve within the range of 0-1000 pg mL⁻¹. One hundred microliters of standards or test samples (serum or cell lysates) were added to antibody-precoated 96-well plates. Blank control wells were included. Plates were sealed and incubated at 37°C for 90 min. After incubation, wells were aspirated and washed five times with 300 μl wash buffer per well, allowing 30 s soaking between washes. Residual liquid was removed by blotting on absorbent paper. One hundred microliters of biotinylated antibody working solution was added to each well except blank controls, followed by incubation at 37°C for 60 min. Wells were washed as described above. Subsequently, 100 μl of TMB substrate solution was added to each well and incubated at 37°C in the dark for 15–30 min until color development was observed. The reaction was terminated by adding 50 μl of stop solution (2 M H_2_SO_4_), resulting in a color change from blue to yellow. OD_450_ was measured within 30 min using a microplate reader. APE1 concentrations in samples were calculated based on the standard curve. All standards and samples were measured in triplicate, and data are presented as mean ± standard deviation.

### Statistical analysis and reproducibility

Statistical analyses were performed using Origin 2024, GraphPad Prism 9.4.1, Microsoft Excel 2016, and ImageJ 1.53. All experiments were conducted with three independent technical replicates (*n* = 3). Detailed information regarding data presentation and sample sizes is provided in the figure legends. No statistical methods were used to predetermine sample size. No data were excluded from the analyses. The experiments were not randomized, and investigators were not blinded to group allocation during experiments or outcome assessment. For bar plots, statistical comparisons among groups were performed using unpaired one-way analysis of variance (ANOVA) followed by Tukey’s multiple comparison test. For fluorescence kinetic curves, data represent the mean of three independent technical replicates and were fitted using a one-phase exponential association model. For violin plots, statistical comparisons between negative and positive patient groups were conducted using unpaired two-tailed Student’s *t*-tests. Fluorescence signals were processed by subtracting the fluorescence intensity of the reporter-only control from the endpoint fluorescence at the indicated time point. The normalized fluorescence for each group was calculated by dividing the background-corrected fluorescence intensity by that of the positive control according to the following formula:


(1) \begin{eqnarray*}
\mathrm{Normalized}{\mathrm{\ }}\mathrm{fluorescence}{\mathrm{\ = \ }}\frac{{F{\mathrm{ - }}{{F}_{{\mathrm{\mathrm{ reporter-only} }}}}}}{{{{F}_{{\mathrm{positive\ control}}}}{\mathrm{ - }}{{F}_{{\mathrm{\mathrm{ reporter-only} }}}}}},
\end{eqnarray*}

where *F* represents the fluorescence intensity of the test sample at the specified time point, and ${{F}_{{\mathrm{reporter - only}}}}$ denotes the fluorescence signal measured in the presence of FQ-reporter alone.

The limit of detection (LOD) was defined based on the fluorescence signal obtained from the no-target control. The LOD threshold was calculated as the mean fluorescence intensity of reactions performed in the absence of target plus three times the standard deviation (SD) (*n* = 3):


(2) \begin{eqnarray*}
{\mathrm{LOD}} = {{F}_{\mathrm{ no}\ \mathrm{target}}} + \left({\mathrm{3 \times SD}}\right),
\end{eqnarray*}

where *F*_no target_ represents the fluorescence intensity measured in the complete reaction system without target.

For calculation of enzymatic reaction kinetics, the fluorescence signal was calibrated using a standard curve generated in this study (Supplementary Fig. S4). Briefly, the background-subtracted fluorescence intensity at a given time point *t* was converted into the concentration of cleaved substrate by dividing the signal by the slope of the calibration curve (Supplementary Fig. S4). The initial reaction velocity V_0_ was then calculated. Enzyme kinetic parameters were obtained by fitting the data to the Michaelis–Menten equation under a total concentration of catalytically active Cas13a-crRNA RNP (E_t_) of 1 nM.


(3) \begin{eqnarray*}
\mathrm{Cleaved}{\mathrm{\ }}\mathrm{substrate}{\mathrm{\ (}}\mathrm{ nM}{\mathrm{)\ = \ }}\frac{{{{F}_t}{\mathrm{ - }}{{F}_{\mathrm{reporter-only}}}}}{{\mathrm{slope}}},
\end{eqnarray*}

(4) \begin{eqnarray*}
{{V}_{\mathrm{0}}}{\mathrm{\ = \ }}\frac{{\mathrm{Cleaved}{\mathrm{\ }}\mathrm{substrate}{\mathrm{\ (}}\mathrm{ nM}{\mathrm{)}}}}{{t{\mathrm{\ (}}s{\mathrm{)}}}}.
\end{eqnarray*}

## Results

### Noncontiguous DNA requires positional overhangs to activate LbuCas13a

**Figure 1.**
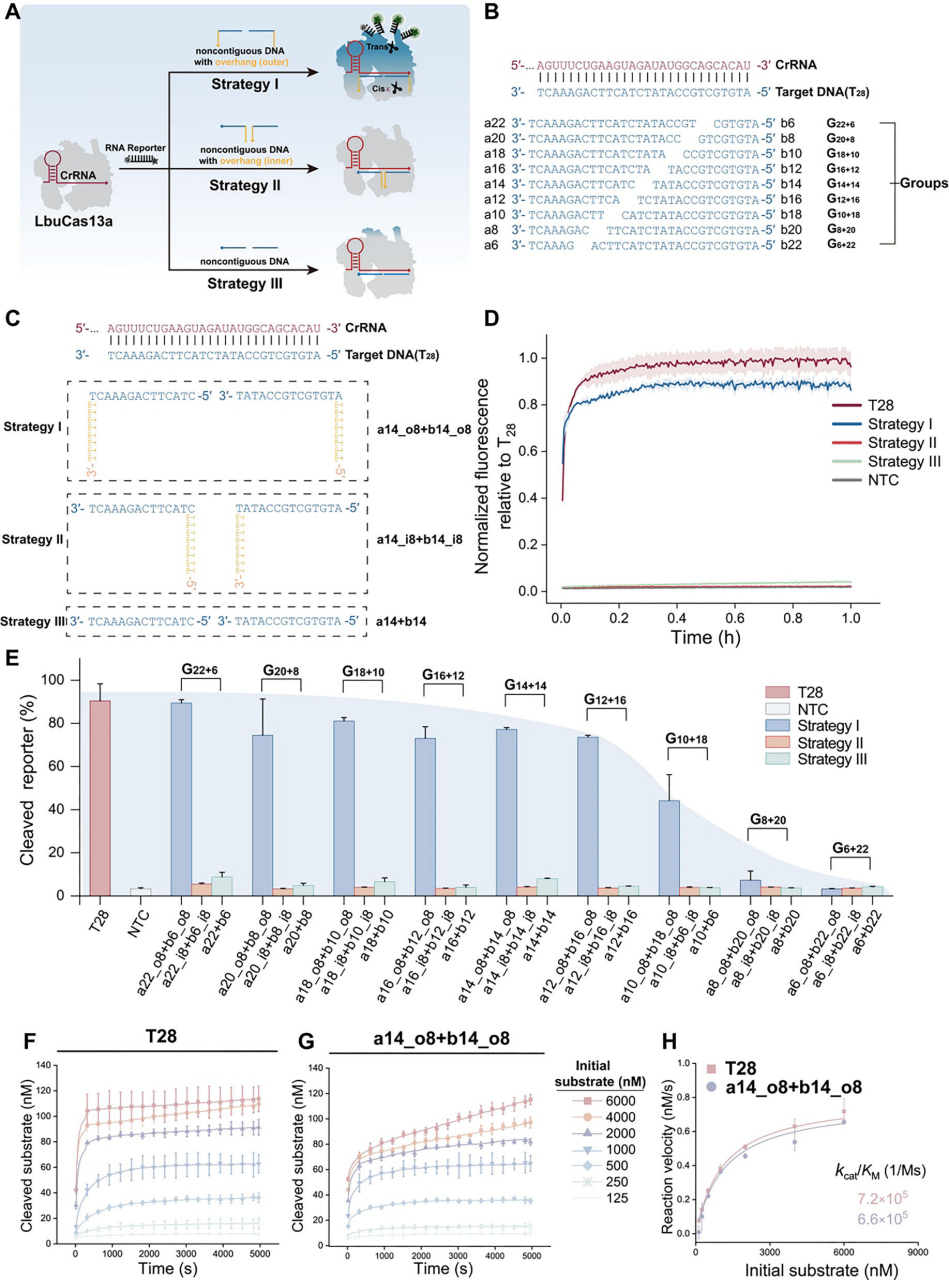
Noncontiguous DNA requires positional overhangs to activate LbuCas13a. (**A**) A schematic diagram of three strategies for activating LbuCas13a. (**B**) A schematic diagram of nine groups of target DNAs (G_22-6_ to G_6-22_). The crRNA guide region and target DNA strands are labeled in the schematic. (**C**) Sequences of G_14–14_ target DNAs used in Strategy I, Strategy II, and Strategy III. (**D**) Fluorescence analysis of LbuCas13a activity triggered by G_14-14_ target DNAs. (**E**) Relative *trans-*cleavage activity of LbuCas13a activated by G_22-6_ to G_6-22_ target DNAs. NTC and T28 are used as negative and positive controls, respectively. (**F**) *trans-*cleavage progress curves for LbuCas13a activated with the T28. (**G**) *trans-*cleavage progress curves for LbuCas13a activated with Strategy I (a14_o8 + b14_o8). Data in panels (F) and (G) are obtained at a fixed activated enzyme concentration of 1 nM. (**H**) Initial reaction velocity versus substrate concentration for T28 and a14_o8 + b14_o8. A nonlinear fit to a Michaelis–Menten curve was performed to obtain (for T28 and a14_o8 + b14_o8, respectively) *k*_cat_ = 0.80 and 0.77 s^−1^ and *K_M_* = 1119 and 1168 nM, respectively. Data are presented as mean ± s.d. (*n* = 3 independent experiments).

To systematically explore the activation behavior of LbuCas13a by noncontiguous DNA targets, three strategies were developed (Fig. 1A). Strategy I consists of two DNA strands bearing outer overhangs, in which the blue complementary regions pair with the spacer region of the crRNA. Strategy II retains identical complementarity but positions the overhangs internally. Strategy III lacks overhang structures and relies on two DNA strands that contiguously pair with the crRNA spacer region. Previous research has systematically investigated the impact of DNA target length on LbuCas13a activation, revealing a significant decline in *trans-*cleavage activity when the target length is reduced to 22-nt or fewer [12, 25]. Based on this established activation threshold, we began designing from a complete complementary strand of 22 nucleotides and generated noncontinuous constructs by systematically adjusting the lengths of the complementary strands. Specifically, the lengths of the two complementary segments were adjusted in 2-nt increments across the spacer region, generating nine groups of noncontiguous DNA targets (G_22–6_ to G_6–22_) (Fig. 1B and Supplementary Table S1). Each group contained targets corresponding to Strategy I, Strategy II, and Strategy III, with identical complementary strand length combinations. To ensure comparability among different architectures, we selected the centrally distributed G_14–14_ group as a representative example for initial evaluation. In this group, Strategy I included a14_o8 and b14_o8, Strategy II included a14_i8 and b14_i8, and Strategy III comprised a14 and b14 (Fig. 1C).

Fluorescent RNA cleavage assays were used to assess the *trans-*cleavage activity of LbuCas13a after activation via DNA. Under identical experimental conditions, strategies II and III were unable to efficiently activate *trans*-cleavage, producing a near-background fluorescence signal. In contrast, Strategy I strongly activated the nuclease activity of LbuCas13a, with cleavage rates comparable to those produced by the continuous DNA target (T28) (Fig. 1D). These striking differences suggest that the spatial arrangement of noncontinuous DNA greatly influences LbuCas13a activation. The outer overhang configuration of Strategy I is thought to promote the spatial co-presence of complementary segments within a confined local environment, thus facilitating the formation of an activating complex. Conversely, the inner overhang of Strategy II and the completely isolated configuration of Strategy III may inhibit this spatial coordination, potentially hindering stable activation. We further investigated whether the nucleotide composition of the outer overhang influences activation efficiency. Substitution with A, C, G, or T outer overhang sequences did not show significant differences in activation rate (Supplementary Fig. S1). Therefore, for consistency, poly-T outer overhangs were used in subsequent experiments. Furthermore, typical fluorescence evolution curves over time were obtained for all target groups from G_22-6_ to G_6-22_ (Supplementary Fig. S2). Strategy I consistently generated detectable activation signals across a wide range of complementary length combinations, maintaining relatively high activation efficiency in the intermediate group, with a notable inflection point around G_10-18_, followed by a sharp decline (Fig. 1E). In contrast, strategies II and III showed significantly reduced activity, almost no activity in some length combinations..

We next quantified the kinetic parameters of LbuCas13a activated by the T28 and by Strategy I (a14_o8 + b14_o8). Fluorescence *trans-*cleavage reactions were measured across reporter concentrations ranging from 125 to 6000 nM in the presence of 1 nM activator (Fig. 1F and G, and Supplementary Fig. S3). A fluorescence intensity–concentration calibration curve was constructed prior to kinetic analysis (Supplementary Fig. S4) and used for signal correction. Initial reaction velocities were fitted to the Michaelis–Menten model to estimate *k*_cat_ and *K_M_* values for both activation conditions (Fig. 1H). The resulting apparent second-order rate constants (*k*_cat_/*K_M_*) were on the order of 10^5^ M^−1^ s^−1^, approximately one order of magnitude lower than certain previously reported values [26, 27], which may reflect differences associated with DNA-mediated activation. Notably, under identical experimental conditions, the apparent second-order rate constants for Strategy I and the contiguous target were comparable, indicating that Strategy I induces *trans-*cleavage efficiency similar to that achieved by the fully contiguous DNA target.

### Positional and length-dependent overhangs govern LbuCas13a activity

**Figure 2.**
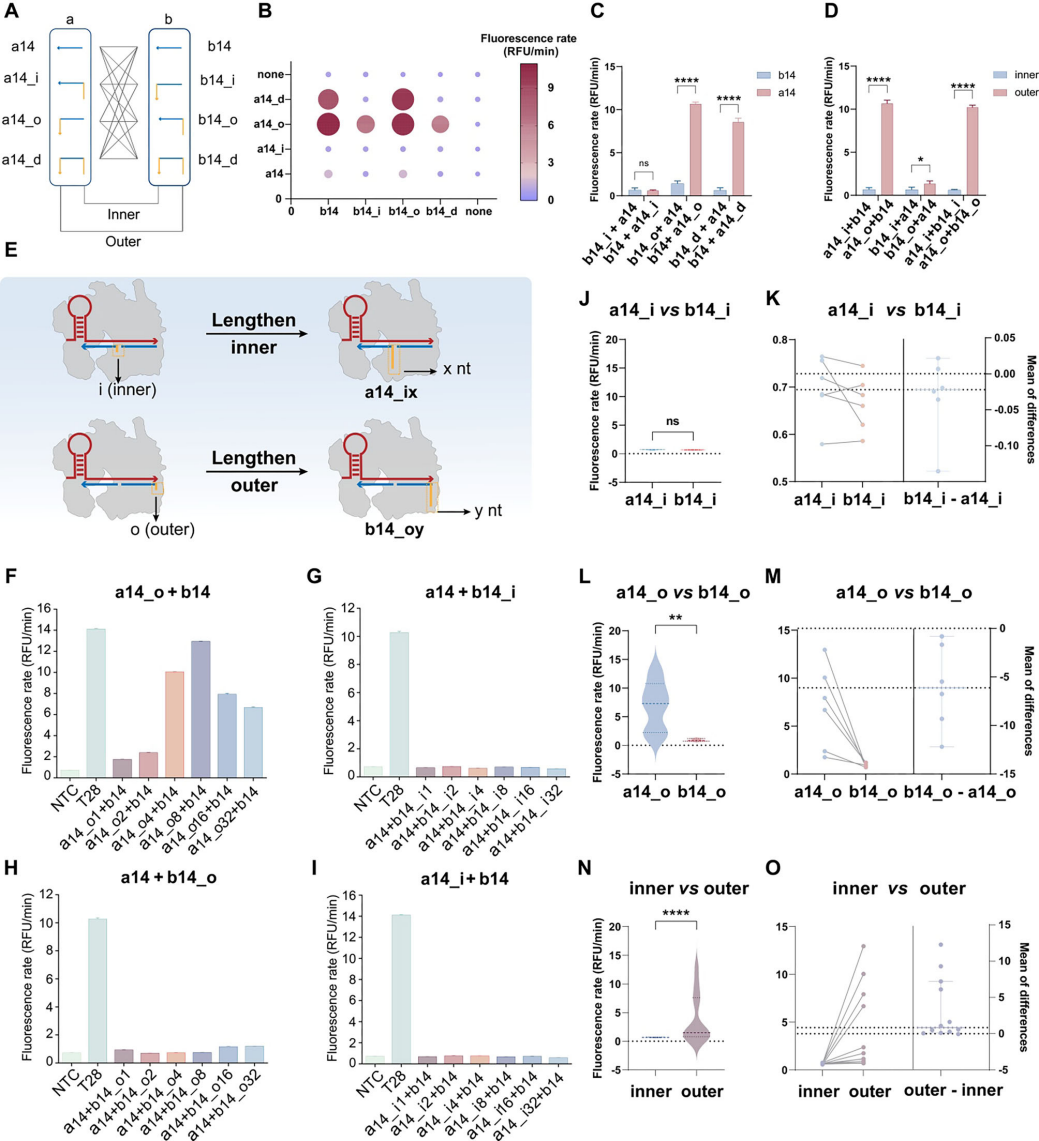
Positional and length-dependent overhangs govern LbuCas13a activity. (**A**) A schematic diagram of the positional combination pattern of noncontiguous DNA architectures. (**B**) Bubble plot showing the positional combination patterns of noncontinuous DNA architectures. Both bubble size and color intensity represent the fluorescence rate, which was calculated from the slope of the fluorescence curves. (**C**) Comparison of a and b with different overhang positions. (**D**) Comparison of inner and outer overhang positions with different activators. (**E**) A schematic diagram of overhang at different lengths and positions. (**F**–**I**) Fluorescence rates of overhangs of different lengths at four different positions. (**J, K**) Pairwise comparison of the inner overhang lengths of a and b. (**L, M**) Pairwise comparison of the outer overhang lengths of a and b. (**N, O**) Pairwise comparisons of inner and outer overhang. Two-tailed paired *t*-tests were used for statistical difference analysis. *P-value <.05; **P-value <.01; ***P-value <.001; ****P-value <.0001. Data are presented as mean ± s.d. (*n* = 3 independent experiments).

To systematically evaluate how overhang structures influence LbuCas13a activation in noncontiguous DNA architectures, we first classified activators based on the position of the overhang relative to the cleavage site into inner overhang (a-i/b-i), outer overhang (a-o/b-o), and dual overhang configurations containing both inner and outer overhangs (a-d/b-d) (Fig. 2A). By combinatorially introducing these overhang types into the a and b modules, we constructed 16 noncontiguous DNA configurations covering all possible spatial arrangements to assess positional effects on LbuCas13a activation (Fig. 2A). To eliminate sequence-dependent variability, all overhangs were designed as 8-nt polyT sequences. Bubble plot analysis further showed that the spatial distribution of overhangs markedly affected LbuCas13a *trans*-cleavage activity; in this plot, both bubble size and color intensity represent the fluorescence rate calculated from the slope of the fluorescence curves (Fig. 2B). The presence of an outer overhang in the a module had a strong impact on the performance: when the a module lacked an outer overhang (e.g. a14 and a14_i), fluorescence signals remained at background levels, whereas configurations retaining the a-module outer overhang exhibited detectable *trans-*cleavage activity (e.g. a14_o and b14_i). Further analysis indicated that the inner overhang in the b module functioned as a limiting factor for activation. In configurations where the a module contained both outer and inner overhangs, activation was maintained provided the b module lacked an inner overhang; however, introduction of an inner overhang into the b module reduced fluorescence signals to near-background levels (Fig. 2B). In contrast, introducing an outer overhang solely into the b module did not produce activation comparable to that observed with the a-module outer overhang (Supplementary Figs S5 and S6). Collectively, these observations indicate that inner overhangs are generally associated with loss of activity, whereas the functional outcome of outer overhangs depends on module identity (Fig. 2C and D).

Having established the positional dependence of overhang-mediated activation, we next examined the effect of overhang length under defined spatial configurations. We systematically extended the length of inner or outer overhangs in the a or b module (Fig. 2E). Here, a14_ix represents an inner overhang of x nt in the a module, whereas b14_oy represents an outer overhang of y nt in the b module. In the a-module outer overhang configuration, LbuCas13a *trans-*cleavage fluorescence rates varied with overhang length, reaching maximal activation at 8 nt, followed by a decline at longer lengths (Fig. 2F and Supplementary Fig. S7A). In contrast, in the b-module outer overhang configuration, fluorescence rates increased progressively with overhang length within the tested range and did not exhibit a detectable decrease (Fig. 2H and Supplementary Fig. S8A). For inner overhang configurations, no detectable LbuCas13a activation was observed regardless of overhang length or module identity (Fig. 2G–I and Supplementary Figs S7B and S8B), consistent with the positional analysis described above. These results indicate that overhang length influences activation only within spatial configurations permissive for LbuCas13a activity.

To compare the effects of position and modulus without the effect of length, we made pairwise comparisons using the same overhang lengths. In the inner overhang condition, groups a_i and b_i showed no significant difference, and both stayed at the background level (Fig. 2J and K). But in the outer overhang condition, groups a_o and b_o showed significant differences (Fig. 2L and M). Activation was detected only in the a_o configuration. We then compared the inner and outer overhang groups at the same length. Activation was still observed only in the outer overhang configuration (Fig. 2N and O).

### Molecular dynamics simulations of binding differences between LbuCas13a and DNA with distinct overhang structures

**Figure 3.**
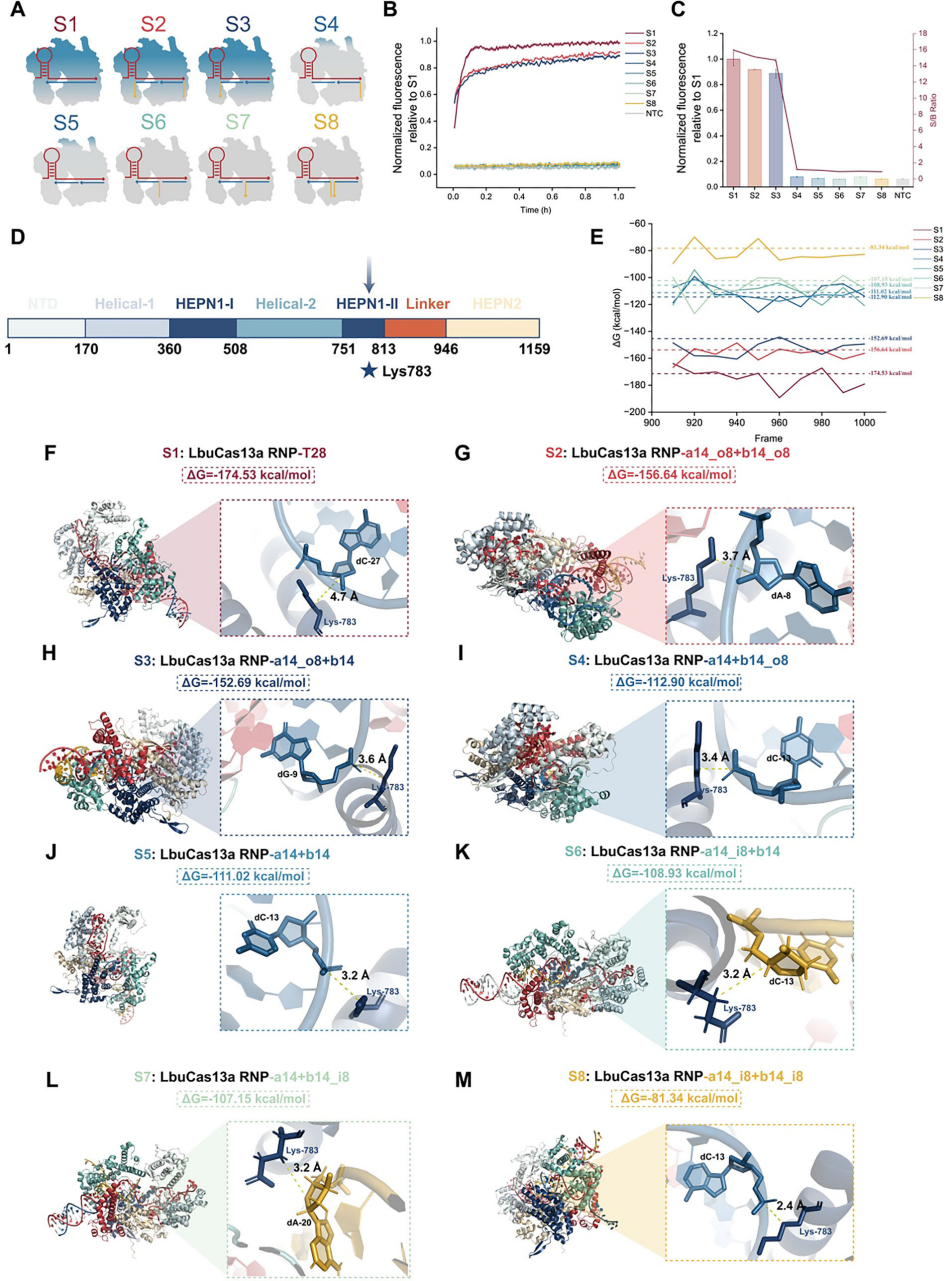
MD simulations revealed the differences in binding between LbuCas13a and different DNA structures. (**A**) A schematic diagram of the binding of eight different DNA structures (S1–S8) to LbuCas13a. S1 corresponds to the adjacent T28 DNA target, while S2–S8 represent non-adjacent DNA structures containing outer or inner overhangs. (**B**) Fluorescence kinetics of LbuCas13a *trans*-cleavage activity triggered by S1–S8, with NTC as the negative control. (**C**) Comparison of fluorescence intensity and signal-to-background (S/B) ratios for S1–S8. Data are presented as mean ± s.d. (*n* = 3 independent experiments). (**D**) Domain architecture of LbuCas13a. Structural domains include NTD, Helical-1, HEPN1, Helical-2, HEPN1-II, Linker, and HEPN2. The position of Lys783 within the HEPN1-II region is indicated. (**E**) Comparison of binding free energies (ΔG) between LbuCas13a and S1–S8 calculated using the MM/GBSA method. (**F**–**M**) Predicted structural models of LbuCas13a RNP in complex with S1–S8, respectively. Enlarged views show the local stick representation highlighting the spatial distance between Lys783 in the REC domain and the bound DNA.

To systematically investigate the structural requirements for LbuCas13a activation, we designed a series of noncontinuous DNA architectures (S2–S8) based on the contiguous target T28 (S1), with distinct overhang configurations (Fig. 3A). Real-time fluorescence kinetics results showed that S2 and S3 maintained significant fluorescence signals compared to S1, while the responses of S4-S8 were close to background levels (Fig. 3B). The signal-to-background (S/B) and fluorescence output of structures S1–S3 were both higher than those of S4-S8 (Fig. 3C). To elucidate the structural basis of these differences, we performed molecular modeling and MD simulations. Structural models of the binding of the LbuCas13a RNP complex with S1–S8 were constructed using AlphaFold3 (Fig. 3F–M). Binding free energy calculations (Fig. 3E) suggested significant differences between the different DNA structures, with S1 exhibiting the lowest binding free energy, while structures with flexible ends showed decreased binding affinity. This trend is consistent with the fluorescence measurements. Energy breakdown per residue (Supplementary Figs S10–S13) and the total free energy difference between the bound and unbound states (Supplementary Table S2) further support the structure-dependent energy variation.

At atomic resolution, we investigated the centroid distance between Lys783 and DNA in various DNA structures. Lys783 is located within the critical loop of the HEPN1-II domain and deep within the DNA binding groove (Fig. 3D). The average Lys783-DNA distances from S1 to S8 were 4.7, 3.7, 3.6, 3.4, 3.2, 3.2, 3.2, and 2.4 Å, respectively (Fig. 3F–M). These distances do not exhibit a simple linear relationship with the binding free energy but rather reflect the geometric differences in the deep regions of the binding groove occupied by different DNA structures. Larger distances generally correlate with geometrically relaxed binding states stabilized by distributed interactions, while smaller distances indicate localized geometric compression due to structural mismatch. These observations suggest that Lys783, as a geometrically sensitive site, reflects spatial adaptability rather than directly determining binding strength.

MD simulations further supported structure-dependent differences in complex stability and flexibility. RMSD analysis showed that S1 rapidly converged and maintained the lowest fluctuation, whereas S8 exhibited greater structural variation; S2–S7 showed intermediate stability (Supplementary Fig. S9). Consistently, RMSF analysis revealed lower residue flexibility in S1 and increased flexibility in the DNA-binding region of S8. Notably, the S1 architecture increased the distance between the Helical-1 and HEPN2 domains while maintaining lower fluctuation, consistent with a more stable inter-domain arrangement. Additional analyses of the Rg, intermolecular hydrogen bonding, and SASA further supported a more compact and stable binding interface for S1, whereas S8 displayed reduced structural complementarity and greater conformational variability (Supplementary Figs S14–S16).

Experimental results together with molecular modeling and MD simulations support the hypothesis that noncontinuous DNA activation of LbuCas13a requires specific structural adaptation conditions. Not all DNA structures with sticky ends were associated with efficient activation. DNA structures that maintain favorable spatial orientation at the putative activation interface (e.g. S2–S3) are more likely to provide sufficient conformational constraints and interfacial complementarity to support efficient activation. In contrast, geometrically incompatible structures (e.g. S4–S8) may be less capable of maintaining stable protein–DNA interactions, which is consistent with their reduced activation efficiency observed experimentally.

### Noncontiguous ssDNA with outer overhangs enhances single-nucleotide discrimination by LbuCas13a

**Figure 4.**
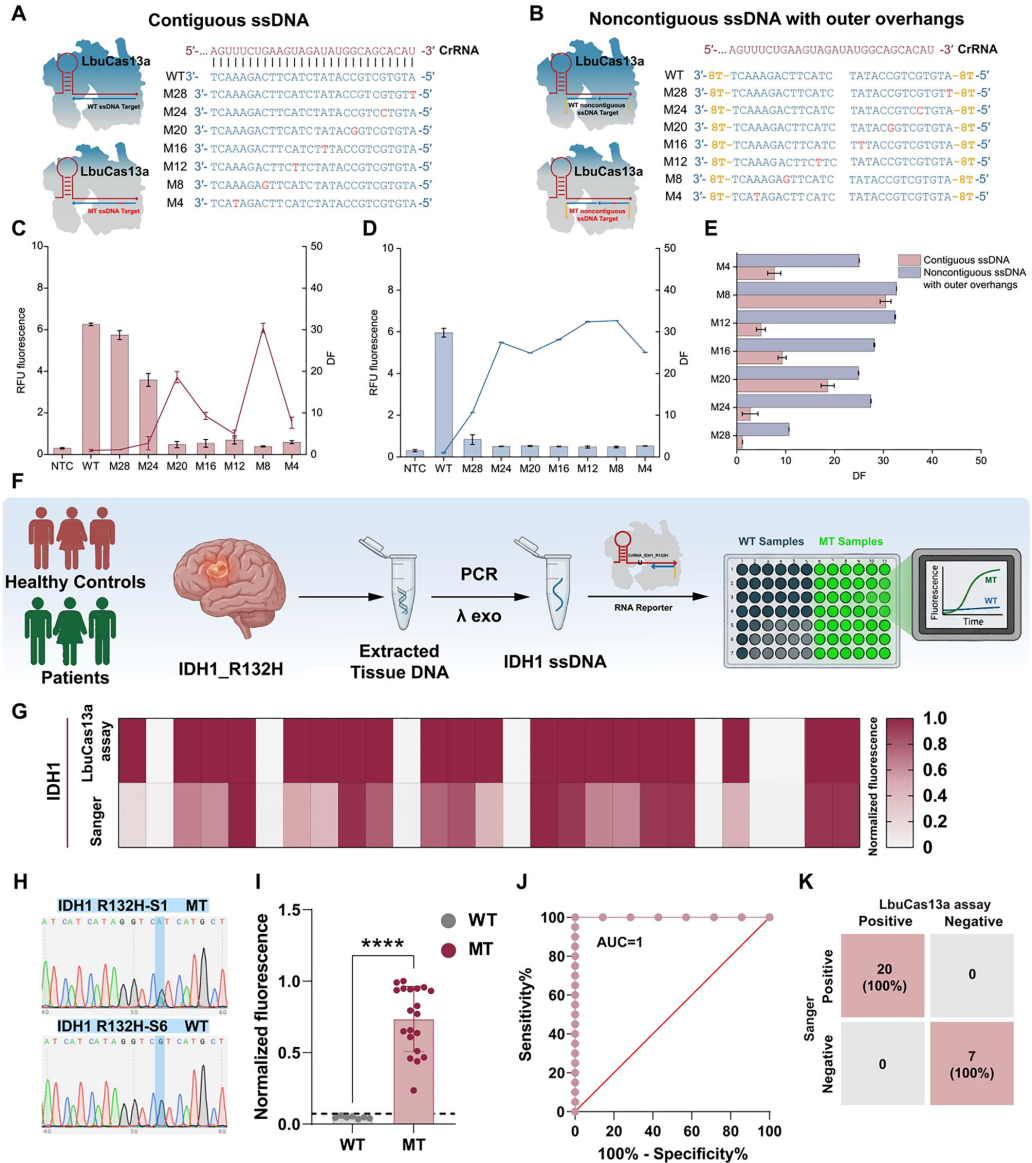
Noncontiguous ssDNA with outer overhangs enhances single-nucleotide discrimination by LbuCas13a and enables IDH1 R132H detection in clinical samples. (**A**) A schematic diagram of single-nucleotide mismatch discrimination using contiguous ssDNA targets. Single-base mismatches were introduced at different positions in the WT target. (**B**) A schematic diagram of single-nucleotide mismatch discrimination using noncontiguous ssDNA with outer overhangs. Single-base mismatches were introduced at different positions in the WT target. (**C**) Fluorescence signals and corresponding WT/MT DF for contiguous ssDNA targets containing mismatches at different positions. NTC, no-target control. (**D**) Fluorescence signals and corresponding WT/MT DF for noncontiguous ssDNA with outer overhangs containing mismatches at different positions. NTC, no-target control. (**E**) Comparison of DF values between contiguous ssDNA and noncontiguous ssDNA with outer overhangs at different mismatch positions. (**F**) Workflow for IDH1 R132H detection in clinical tissue samples. Tissue DNA was extracted, PCR-amplified, digested with λ exonuclease to generate ssDNA, and analyzed using the LbuCas13a system. (**G**) Heatmap comparing results from the LbuCas13a assay and Sanger sequencing in clinical samples. (**H**) Representative Sanger sequencing traces of mutant (MT) and wild-type (WT) samples. (**I**) Binary discrimination of the IDH1 R132H mutation based on normalized fluorescence signals. The horizontal dashed line indicates the classification threshold, defined as the mean normalized fluorescence signal of the WT control plus three standard deviations (0.0725). Samples with fluorescence signals above the threshold were classified as IDH1 R132H-positive. ****, *P* < .0001. (**J**) Receiver operating characteristic (ROC) curve for distinguishing WT and MT clinical samples. (**K**) Confusion matrix comparing this method with Sanger sequencing (*n* = 27).

Single-base mismatch tolerance remains a limitation in CRISPR-based nucleic acid recognition [25, 28, 29], particularly when continuous targets form stable crRNA-target duplexes. We therefore asked whether noncontiguous DNA architectures could impose a stricter activation threshold and improve single-nucleotide discrimination. To test this hypothesis, we constructed contiguous ssDNA and noncontiguous ssDNA with outer overhangs bearing single-nucleotide mismatches at positions 4–28, and compared their effects on LbuCas13a *trans-*cleavage activity at 10 nM target concentration (Fig. 4A and B). Contiguous ssDNA showed only limited and position-dependent discrimination, with clear WT/MT separation observed mainly at positions 8 and 20, whereas mismatches at positions 4, 12, 16, 24, and 28 were more tolerated (Fig. 4C). In contrast, noncontiguous ssDNA with outer overhangs exhibited high discrimination across positions 4–24, with only position 28 showing weaker separation (Fig. 4D). Notably, positions 4, 12, and 24, which were poorly resolved in the contiguous system, were markedly improved in the noncontiguous system. This may be because, at these positions, the outer-overhang ssDNA contains fewer interacting residues than full-length ssDNA (Supplementary Fig. S10). Overall, whereas contiguous ssDNA displayed strong positional dependence, noncontiguous ssDNA with outer overhangs maintained higher discrimination across a broader range of sites (Fig. 4E), supporting its utility for highly specific mutation recognition.

Next, we tested this strategy using clinical tissue samples. We focused on the hotspot single-nucleotide variant IDH1 R132H in glioma. We collected 27 postoperative glioma tissue samples from the First Affiliated Hospital of Chongqing Medical University (Fig. 4F and Supplementary Table S3). Genomic DNA was extracted, amplified by PCR, and digested with an exonuclease to generate single-stranded DNA for LbuCas13a-based fluorescence analysis. This method correctly identified 20 IDH1 R132H-positive samples and seven negative samples, completely consistent with Sanger sequencing results (Fig. 4G and H, and Supplementary Figs S17 and S18). The normalized fluorescence intensity of the mutant samples was significantly higher than that of the wild-type samples (Fig. 4I). The area under the curve (AUC) value of ROC curve analysis was 1.0 (Fig. 4J), and the sensitivity and specificity of this method relative to Sanger sequencing were both 100% (Fig. 4K). Overall, these results demonstrate that IDH1 R132H can be accurately identified in clinical tissue samples and highlight the potential of this strategy for highly specific mutation analysis..

### LbuCas13a-based detection of APE1 protein biomarker

**Figure 5.**
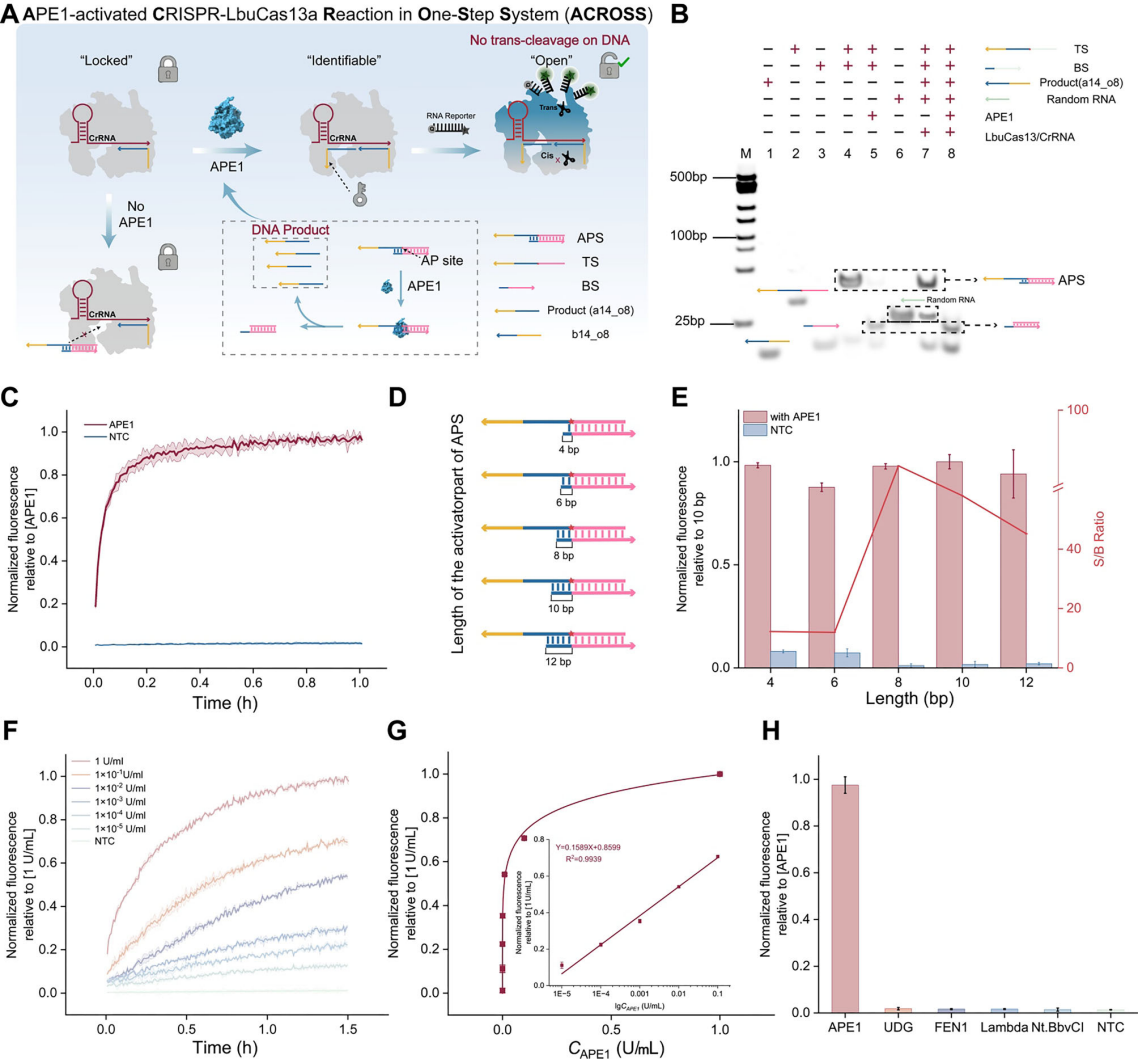
LbuCas13a-based detection of the APE1 protein biomarker. (**A**) A schematic diagram of the APE1-activated ACROSS system, in which APE1-mediated cleavage of the structure-switchable APS releases a14_o8 that cooperatively engages with b14_o8 to activate LbuCas13a *trans-*cleavage. (**B**) PAGE analysis of APS assembly and APE1-triggered activation. Lane M, DNA marker; lane 1, Product (a14_o8); lane 2, TS; lane 3, BS; lane 4, APS; lane 5, APS + APE1; lane 6, random ssRNA; lane 7, APS + LbuCas13a/crRNA + random ssRNA; lane 8, APS + APE1 + LbuCas13a/crRNA + random ssRNA. (**C**) Real-time fluorescence kinetics of the ACROSS system in the presence and absence of APE1. (**D**) Design of BS activator regions with varying complementary lengths (4–12 bp). (**E**) Corresponding fluorescence signals and signal-to-background (S/B) ratios for different BS complementary lengths. (**F**) Real-time fluorescence signals at increasing APE1 concentrations ranging from 1.0 × 10⁻⁵ to 1.0 U mL⁻¹. (**G**) Calibration curve derived from real-time fluorescence intensities in panel (F). The inset shows the linear relationship between fluorescence intensity and logarithmic APE1 concentration. (**H**) Selectivity analysis of the sensing system toward APE1 compared with other nucleases (UDG, FEN1, λ exonuclease, and Nt.BbvCI). Data are presented as mean ± s.d. (*n* = 3 independent experiments).

To evaluate the applicability of the proposed strategy for protein detection, APE1 was selected as a model target owing to its specific cleavage of AP site-containing dsDNA. Leveraging this enzymatic property, we constructed an ACROSS (Fig. 5A). In this design, a structure-switchable APS, composed of an AP-containing target strand (TS) and a blocking strand (BS), maintains a locked conformation in the absence of APE1, thereby preventing Cas13a activation. Upon APE1-mediated cleavage, APS releases the a14_o8 fragment, which co-binds with the pre-loaded b14_o8 and binds to different regions of crRNA, thereby stabilizing the active conformation of LbuCas13a and restoring its *trans-*cleavage activity. Notably, LbuCas13a selectively cleaves RNA without degrading DNA products, thus preserving the trigger structure and improving the efficiency of the one-pot reaction [13, 30–32].Conversely, in the absence of APE1, the structure of APS is restricted, thereby inhibiting LbuCas13a activation.

Next, we confirmed by PAGE that APE1 effectively triggers APS cleavage. As shown in Fig. 5B, lanes 2 and 3 correspond to TS and BS, respectively, and the slower-migrating band in lane 4 confirms APS assembly. Compared to the intact double-stranded DNA control group (lane 5), a significant degradation band appeared after the addition of APE1. Under APE1 activation conditions, the intensity of random single-stranded RNA bands decreased clearly (lane 8 versus lane 6). No change was observed without APE1 (lane 7). This result showed that Cas13a was successfully activated. Fluorescence kinetic analysis also supported this result (Fig. 5C). After confirming effective activation, we optimized the complementary base length between BS and TS. As shown in Fig. 5D and Supplementary Fig. S19, the signal-to-background ratio (S/B) increased as the complementary length increased (Fig. 5E). The highest S/B was observed at 8 nt. The signal then decreased, likely because the double strand became too stable and limited activator release. Based on this result, we used a complementary length of 8 nt in the following experiments.

Under the optimized conditions, the system showed a concentration-dependent fluorescence response to APE1 (Fig. 5F). Linear regression analysis (Fig. 5G, inset) yielded *Y* = 0.1589 lgC + 0.8599 (*R*² = 0.9939) across a dynamic range of 1.0 × 10^−5^–1.0 × 10⁻¹ U mL^−1^. This result showed that the system could quantify APE1 reliably.The analytical performance of the ACROSS platform was further compared with those of previously reported APE1 detection methods, as summarized in Supplementary Table S4. Selectivity analysis using UDG, FEN1, λ exonuclease, and Nt.BbvCI showed only weak signals compared with APE1 (Fig. 5H and Supplementary Fig. S20). This result showed good specificity.

### ACROSS for intracellular activity imaging

Precise characterization of intracellular APE1 activity is crucial for understanding tumor progression and optimizing risk stratification. As the rate-limiting enzyme in the base excision repair pathway, APE1 maintains genomic integrity and is frequently upregulated in a variety of malignancies [33–36]. Since APE1 function depends not only on expression levels but also on spatial distribution and dynamic enzyme activity, activity-resolved imaging methods are needed. Existing “always-on” probes often generate background signals in non-target cells, limiting the contrast between tumor and normal cells in the complex intracellular environment.

**Figure 6.**
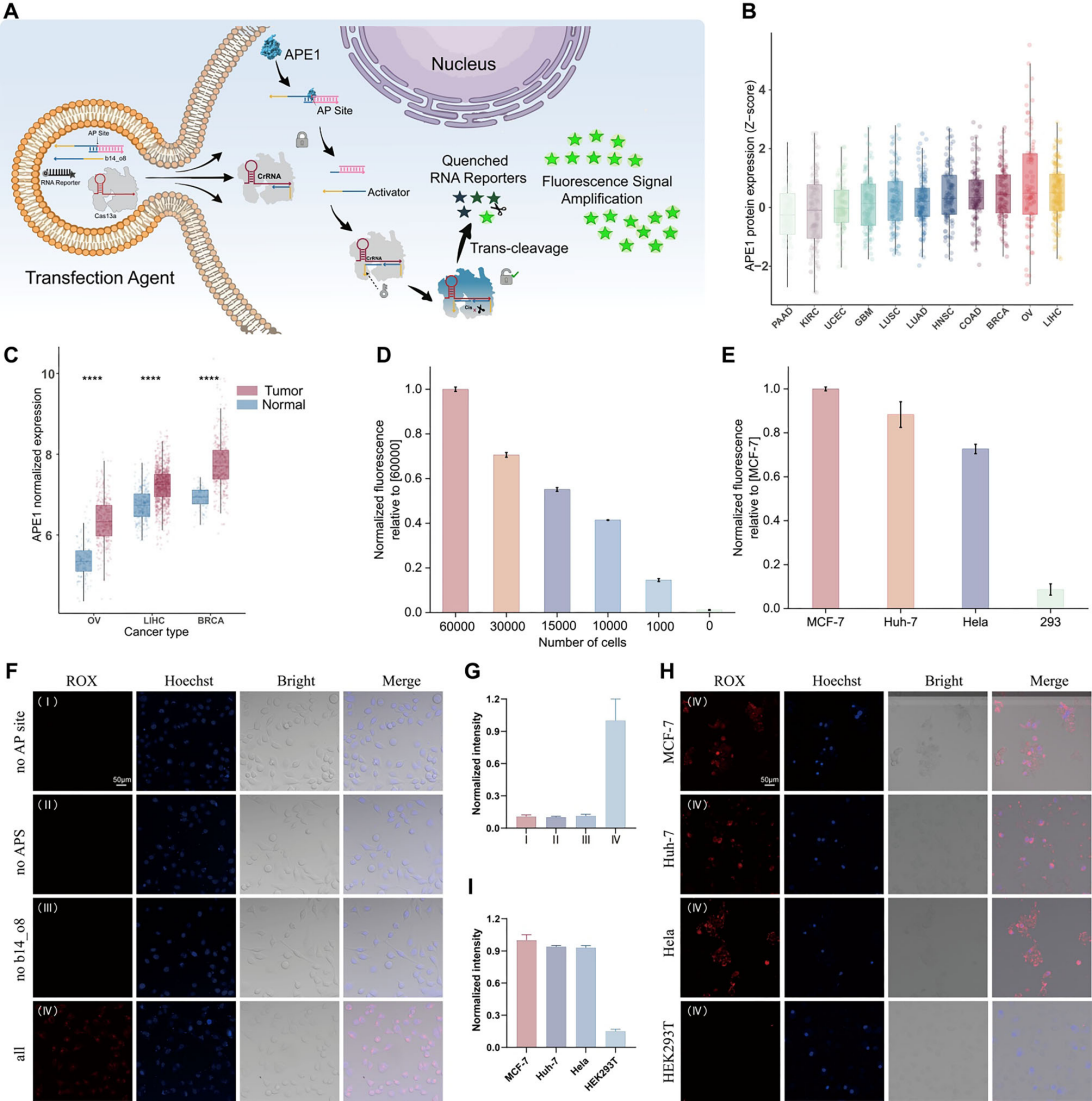
ACROSS for intracellular activity imaging. (**A**) A schematic diagram of the ACROSS system used for APE1 imaging in live cells. The ACROSS system components (LbuCas13a RNP, APS, b14_o8, and the FQ reporter gene) were transfected into cells using Lipofectamine 3000. Intracellular APE1 cleaves the AP site within the APS, triggering structural rearrangement and releasing the activation strand. Activated LbuCas13a subsequently mediates *trans-*cleavage of the FQ reporter gene RNA, generating a fluorescent signal. (**B**) Normalized APE1 protein expression levels across multiple cancer types obtained from Clinical Proteomic Tumor Analysis Consortium database. Elevated expression is observed in liver hepatocellular carcinoma (LIHC), ovarian cancer (OV), and BC. Each dot represents an individual patient sample. The center line indicates the median, boxes represent the interquartile range, and whiskers denote minimum and maximum values. (**C**) Normalized APE1 mRNA expression levels in normal tissues and representative tumor cell lines. Each dot represents an individual sample (*N* = 88, 417, 159, 368, 130, 281, and 1081, respectively). Statistical significance was determined using two-tailed *t*-tests (*P-value <.05; **P-value <.01; ***P-value <.001; ****P-value <.0001). (**D**) Fluorescence responses of ACROSS to lysates derived from increasing numbers of HeLa cells. (**E**) Fluorescence responses of ACROSS to lysates from four human cell lines at equal cell numbers (1 × 10^4^ cells). (**F**) Confocal fluorescence images of Huh-7 cells treated with Group I, Group II, Group III, and Group IV conditions as defined in the main text. (**G**) Quantification of normalized fluorescence intensities corresponding to panel (F). (**H**) Confocal fluorescence images of MCF-7, Huh-7, HeLa tumor cell lines, and HEK293T cells treated with Group IV. (**I**) Quantification of normalized fluorescence intensities corresponding to panel (H). Data are presented as mean ± s.d. (*n* = 3 independent experiments). Scale bars, 50 μm.

To overcome this limitation, we extended our previously established ACROSS system to intracellular imaging, enabling selective visualization of APE1 activity in tumor cells. This system is independent of exogenous stimuli; instead, an increase in endogenous APE1 activity acts as a trigger. As illustrated in Fig. 6A, intracellular APE1 cleaves APS, inducing structural rearrangement and releasing the activating chain. This activation chain promotes conformational activation of LbuCas13a, initiating RNA *trans-*cleavage of the fluorescent reporter gene, thereby generating amplified signal output. Protein analysis revealed elevated APE1 levels in multiple tumor types, including hepatocellular carcinoma, cervical cancer, and BC (Fig. 6B). Elevated APE1 mRNA levels were also observed in three representative tumor cell lines compared to the normal control group (Fig. 6C). Next, we evaluated the response of ACROSS to APE1 derived from cells *in vitro*. As shown in Fig. 6D and Supplementary Fig. S21, fluorescence intensity gradually increased with increasing HeLa cell count, indicating a response to changes in total APE1 content. When using equal cell volumes (1 × 10^4^), lysates from MCF-7, Huh-7 (human hepatocellular carcinoma), and HeLa (human cervical cancer) cells induced different fluorescence outputs (Fig. 6E and Supplementary Fig. S22), indicating differences in the response to APE1 from different cell sources.

Based on these *in vitro* experimental results, we evaluated the application of ACROSS in live-cell imaging. LbuCas13a RNP, APS, b14_o8, and fluorescence quenching reporter (FQ-reporter) genes were transfected into cells using Lipofectamine 3000. Huh-7 cells were divided into four groups: (I) LbuCas13a + FQ-reporter; (II) RNP + b14_o8 + FQ-reporter; (III) RNP + APS + FQ-reporter; and (IV) RNP + APS + b14_o8 + FQ-reporter. Confocal imaging showed negligible fluorescence in group I, indicating reporter stability. Enhanced fluorescence was observed only in group IV, whereas groups II and III showed no detectable signal (Fig. 6F and G), indicating that intracellular signal generation requires the complete activation cascade. We further examined the applicability of ACROSS across different cell types. ACROSS components were transfected into MCF-7, Huh-7, and HeLa cells, with HEK293T (human embryonic kidney cell line) cells as a control. As shown in Fig. 6H and I, strong fluorescence signals were detected in all three tumor cell lines, while no LbuCas13a-mediated fluorescence activation was observed in HEK293T cells. These results indicate that ACROSS can be activated in a variety of tumor cell environments while maintaining low background fluorescence in cells with relatively low APE1 activity.

### ACROSS for clinical sample diagnostics

**Figure 7.**
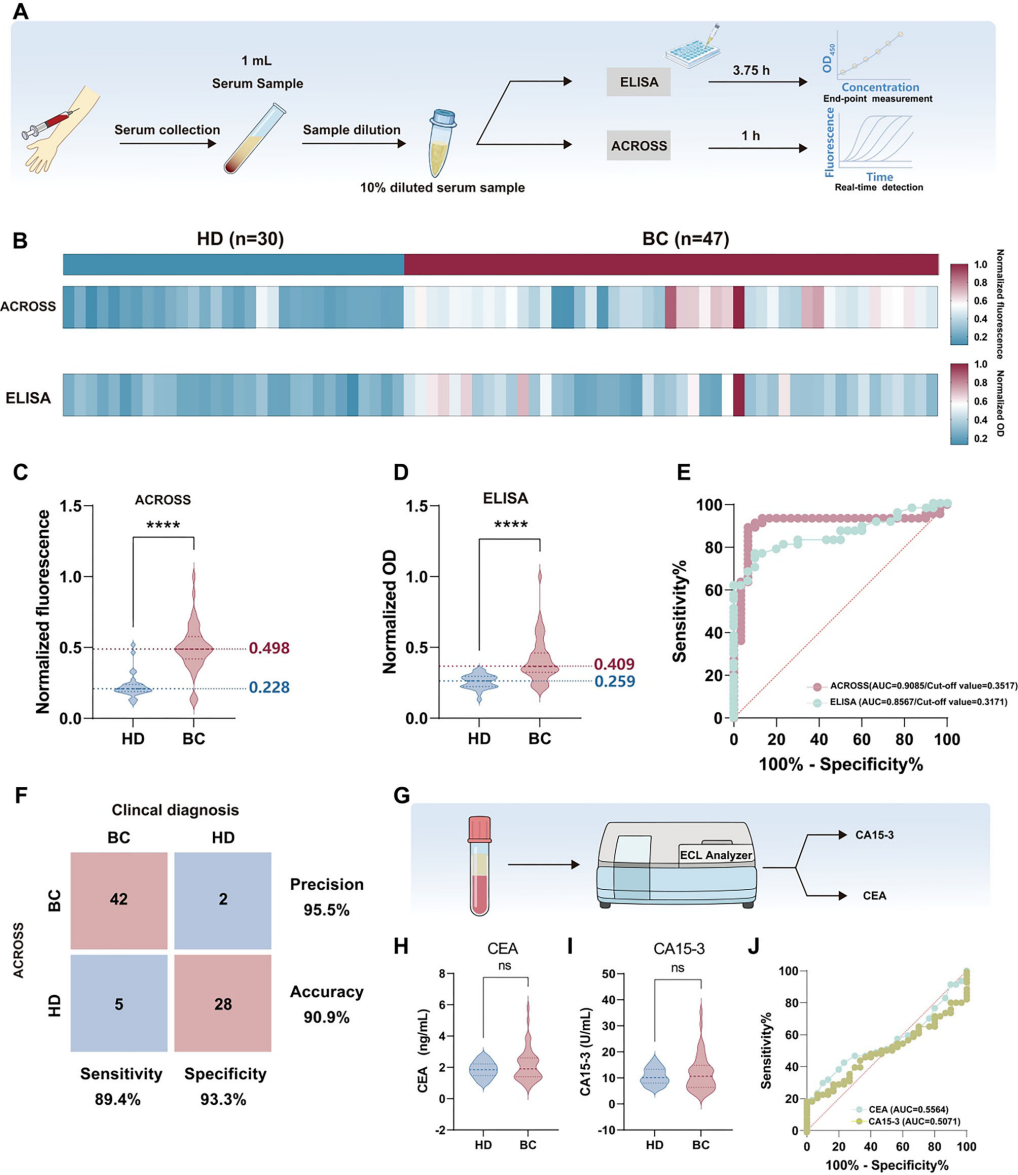
ACROSS for clinical sample diagnostics. (**A**) The diagram illustrates the diagnostic workflow for detecting APE1 in the serum of BC patients and HD using ACROSS and ELISA. Serum samples are diluted to 10% prior to analysis. ELISA detection is achieved through endpoint absorbance, while ACROSS provides real-time fluorescence detection. (**B**) Heatmap visualization of normalized detection signals obtained using ACROSS and ELISA across the HD (*n* = 30) and BC (*n* = 47) cohorts. (**C**) Violin plots showing normalized fluorescence signals measured by ACROSS in HD and BC serum samples. (**D**) Violin plots showing normalized OD values measured by ELISA in HD and BC serum samples. (**E**) ROC curves comparing the diagnostic performance of ACROSS and ELISA for BC detection in serum samples. (**F**) Confusion matrix summarizing the diagnostic performance of ACROSS relative to clinically established diagnoses. Sensitivity, specificity, overall accuracy, and positive predictive value (PPV) are indicated. (**G**) A schematic diagram of clinical detection of BC serum biomarkers CEA and CA15-3. Violin plots with embedded box plots comparing serum levels of CEA (**H**) and CA15-3 (**I**) between HD and BC cohorts. (**J**) ROC curves and corresponding AUC values for CEA and CA15-3 in BC diagnosis. Data are presented as mean ± s.d. (*n* = 3 independent experiments).

Building on the sensitivity and rapid response characteristics of ACROSS, we first validated its feasibility in serum through spike-in recovery experiments (Supplementary Table S5), and then evaluated its performance in clinical serum samples. Serum specimens were collected from 47 patients with BC and 30 HD at the First Affiliated Hospital of Chongqing Medical University (Supplementary Table S6). To minimize matrix interference, all serum samples were diluted to 10% prior to analysis (Fig. 7A). Under identical dilution conditions, APE1 levels were measured using ACROSS and a commercial ELISA kit. Unlike ELISA, which relies on absorbance measurements at the endpoint, ACROSS allows for real-time fluorescence monitoring. The corresponding reaction kinetics for individual patient samples are shown in Supplementary Figs S23–S25. Heatmap visualization of all clinical samples showed a clearer separation between the BC and HD groups using ACROSS, while the ELISA signals showed partial overlap between the groups (Fig. 7B).

Quantitative analysis revealed a significantly higher normalized fluorescence signal in the BC group compared to HD using ACROSS (*P* < .0001), with limited distribution overlap (Fig. 7C). The ELISA also detected increased APE1 levels in the BC samples; however, the normalized OD values ​showed a narrower dynamic range and greater overlap between the groups (Fig. 7D). ROC analysis was performed to compare overall discriminatory performance. ACROSS achieved a higher AUC than ELISA (Fig. 7E). Using optimized threshold values, ACROSS yielded a sensitivity of 89.4%, a specificity of 93.3%, an overall accuracy of 90.9%, and a PPV of 95.5% (Fig. 7F). In the same cohort, ELISA demonstrated a sensitivity of 76.6%, a specificity of 90.0%, an overall accuracy of 81.8%, and a PPV of 92.3% (Supplementary Fig. S26). As an additional point of comparison, two clinically used BC serum biomarkers, carcinoembryonic antigen (CEA), and cancer antigen 15-3 (CA15-3), were measured in the same cohort (Fig. 7G). Neither marker showed a statistically significant difference between the BC and HD groups (Fig. 7H and I). The corresponding ROC analysis showed AUC values ​​close to random classification (Fig. 7J). Overall, under identical sample processing conditions and in the same clinical cohort, ACROSS enabled rapid and stable detection of serum APE1 and demonstrated improved discriminatory performance compared to ELISA, CEA, and CA15-3.

## Discussion

For a long time, the activation mechanism of CRISPR–LbuCas13a has implicitly been based on the assumption of target sequence continuity. In previous studies, continuous complementarity has generally been considered a prerequisite for activation, whether for RNA or DNA substrates, while target sequence length and pairing stability have been considered the main determinants of activity [4, 5, 8, 37]. However, in physiological and engineered environments, Cas13a frequently encounters structured, dynamically rearranged, or spatially separated target sequences. This study systematically investigated whether target sequence continuity constitutes an intrinsic requirement for Cas13a activation by constructing discontinuous DNA structures. We found that even when the total complementarity information meets the reported activation threshold, discontinuous DNA cannot activate Cas13a in the absence of proper spatial organization. These findings indicate that the responsiveness of Cas13a is not solely determined by sequence continuity.

A systematic comparison of different noncontinuous DNA structures revealed a previously unknown principle of structural acceptability. LbuCas13a only enters a stable activated state when noncontinuous DNA fragments are co-presented by appropriately positioned outer overhangs, while inner or misaligned outer overhangs consistently lead to inactivation. Therefore, the function of overhang structures depends not on their length or nucleotide composition, but on whether they meet the spatial requirements for activation. Thus, the conversion of target information into catalytic output depends on structural compliance, rather than simple complementarity accumulation.

MD simulations provide supportive conformational insights into the structural conditions associated with LbuCas13a activation. Binding free energy decomposition suggested that Arg784 is consistently the major energy contributor across the examined DNA structures, consistent with its widespread involvement in the interaction network. However, due to its long and flexible side chains, Arg784 may adopt multiple dynamic contact modes and exhibits energy variability, making it less suitable for distinguishing between structurally compatible and incompatible conformations. Therefore, we focus on Lys783, located deep within the DNA binding groove, as a potential geometric indicator of proper DNA accommodation. In the structurally compatible conformation, DNA appeared to be stabilized through dispersed multi-point interactions without being forced close to Lys783, thus maintaining a relatively relaxed and stable geometry. In contrast, the twisted conformation was associated with localized spatial compression, aberrant contacts, and increased fluctuations around Lys783. Collectively, these observations support the view that LbuCas13a activation depends on appropriate global conformational constraints, rather than isolated strong local interactions.

Therefore, the functional contribution of overhangs is governed primarily by their spatial positioning, whereas overhang length can further modulate activity within permissive configurations and nucleotide composition showed limited effects under the conditions tested. Notably, noncontinuous single-stranded DNA with outer overhangs also expands the single-nucleotide recognition window of LbuCas13a by increasing the structural requirements for effective target recognition. Compared to continuous single-stranded DNA targets, noncontinuous single-stranded DNA with outer overhangs reduces mismatch tolerance over a wider range of locations. We further utilized the same design principle to construct a functional sensor platform. In this system, the protein target produces a noncontinuous DNA product that satisfies the desired spatial conformation, thereby determining whether LbuCas13a enters an activated state. By combining catalytic activity with DNA structural compatibility, this design fundamentally suppresses nonspecific interference. In live-cell imaging, a stable LbuCas13a-mediated signal was observed only in tumor cells with high APE1 activity, while the signal output in control cells was close to background levels. This platform was further applied to serum samples from BC patients, and under standardized conditions, it was able to more clearly distinguish between patient groups and healthy controls compared to conventional ELISA. Importantly, this validation aimed to demonstrate its robustness and reproducibility in complex biological matrices, rather than for diagnostic purposes.

From a broader perspective, the structural permissibility principle described here may provide a useful framework for improving the controllability of Cas13a-based regulation and editing systems. Intracellular Cas13 systems are commonly associated with nonspecific RNA degradation and transcriptional interference [38–40]. Although the present study focused on noncontiguous DNA substrates with outer overhangs, the same design logic may potentially be extended to other structured DNA architectures, such as hairpin, circular, branched, or junction-containing constructs [41–44]. These topologies may impose distinct geometric constraints and modes of target presentation to the LbuCas13a–crRNA complex; however, whether they can support productive activation remains to be determined. More broadly, by restricting catalytic activity to structurally compatible conditions and maintaining the enzyme in an inert state before activation, this strategy can reduce unintended interactions with background RNA. Compared to continuous exposure to the target sequence, structure-guided activation is particularly suitable for applications requiring strict spatiotemporal control, such as conditional RNA editing or programmable transcriptional regulation [45–47]. Although currently at a proof-of-concept stage, regulating Cas activity through spatial and conformational constraints offers a promising direction for improving controllability and minimizing collateral effects.

## Conclusions

In summary, we establish a structural accessibility principle for LbuCas13a activation by noncontiguous DNA. Our results show that activation is not determined only by crRNA complementarity or target continuity. It also requires an overhang-defined DNA architecture that can support a productive LbuCas13a–DNA interface. By using structural design, biochemical validation, and molecular simulations, we show that different overhang topologies have different effects. Some overhang topologies stabilize activation-compatible conformations, while others disturb local accommodation and complex stability. We also show that noncontiguous DNA architectures can improve single-nucleotide discrimination and can be used in an APE1-responsive one-step CRISPR–LbuCas13a sensing platform. LbuCas13a cleaves RNA reporters but does not degrade the DNA-triggering products. As a result, the activating architecture is preserved, and stable signal amplification can be achieved *in vitro*, in live cells, and in clinical samples. Collectively, this work extends structural understanding of Cas13a activation and provides a conceptual framework for developing CRISPR systems with enhanced spatial precision and reduced collateral activity in complex biological environments.

## Supplementary Material

gkag740_Supplemental_File

## Data Availability

The data underlying this article are available in the article and in its online supplementary material.
