# Supplementary material for: A structural accessibility principle for LbuCas13a activation by noncontiguous DNA
Source: Nucleic Acids Res. 2026 Jul 25;54(14):gkag740. doi: 10.1093/nar/gkag740 (PMC13401041; doi:10.1093/nar/gkag740)
Supplement: gkag740_Supplemental_File [file gkag740_supplemental_file.pdf]

# A structural accessibility principle for LbuCas13a activation by noncontiguous DNA

**Weitao Wang[1, 2, †], Yuhan Chen[2, †], Ziyun Li[2, †], Li Zhang[2, †], Kai Gui[2], You Wu[2], Na Yin[2], Xiaole Han[2], Yaoyi Zhang[2], Ruiling Lu[2], Ziheng Zhang[2], Li Wang[3,*], and Guoming Xie[1, 2, *]**

[1]Department of Neurosurgery, The First Affiliated Hospital of Chongqing Medical University, No.1 Youyi Road, Chongqing 40016, P.R China.

[2]Key Laboratory of Clinical Laboratory Diagnostics (Chinese Ministry of Education), College of Laboratory Medicine, Chongqing Medical University, Chongqing, 400016, PR China.

[3]The Center for Clinical Molecular Medical Detection, Engineering Research Center of Chongqing Education Commission of China for IVD Technology Innovation and Translation, Laboratory Medicine Center, The First Affiliated Hospital of Chongqing Medical University, Chongqing 400016, P.R.China.

[*]To whom correspondence should be addressed. Email: guomingxie@cqmu.edu.cn.
Correspondence may also be addressed to Li Wang. Email: leonwong0403@foxmail.com.
[†] These authors contributed equally to this work.

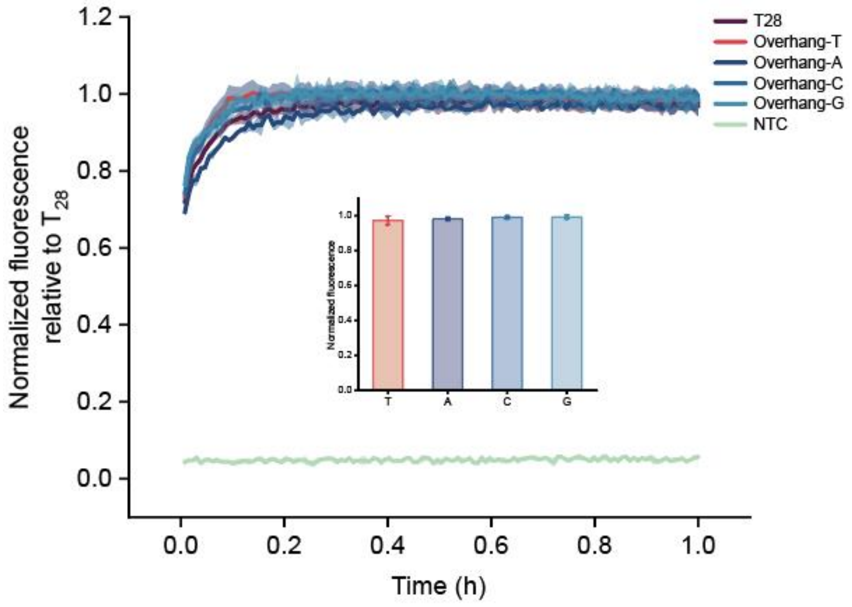


Supplementary Figure S1. Effects of different overhang sequences on *trans*-cleavage of LbuCas13a. Data are presented as mean ± s.d. (n = 3 independent experiments)

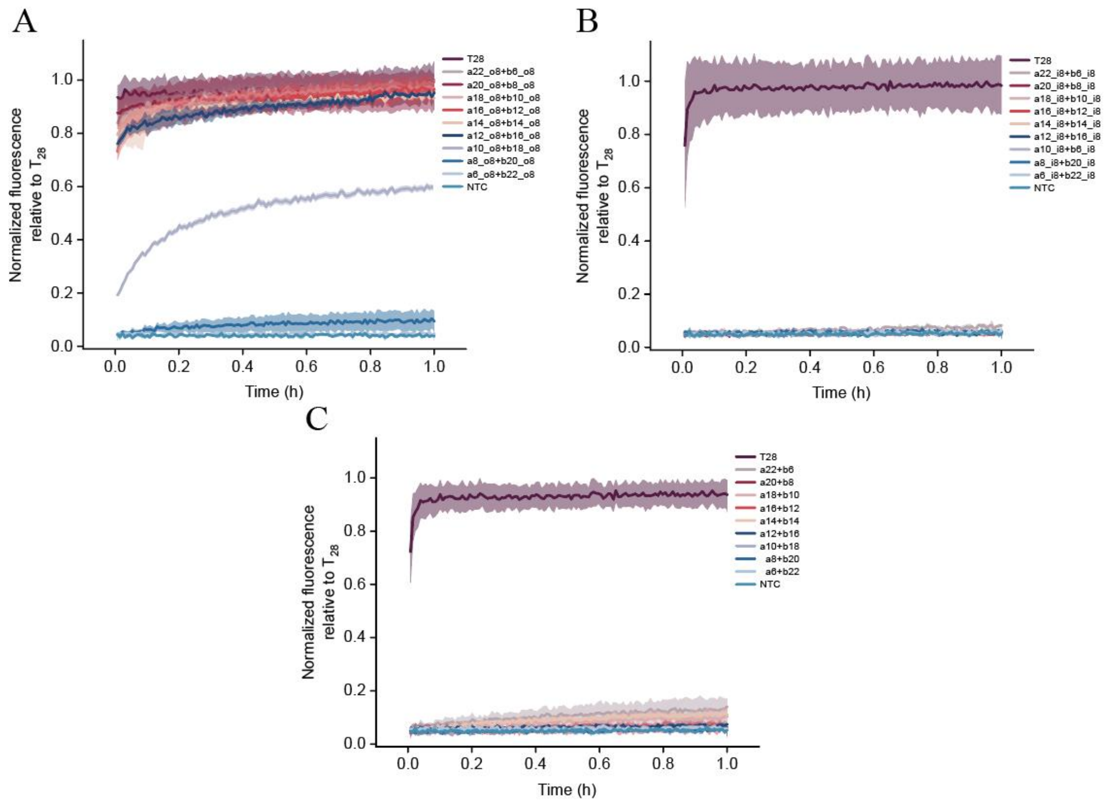


Supplementary Figure S2. Fluorescence kinetics of LbuCas13a activation by different noncontiguous DNA architectures. (A) Quantitative fluorescence signals of CRISPR-Cas13a activation mediated by noncontiguous DNA with dual outer overhangs. (B) Quantitative fluorescence signals of CRISPR-Cas13a activation mediated by noncontiguous DNA with dual inner overhangs. (C) Quantitative fluorescence signals of CRISPR-Cas13a activation mediated by noncontiguous DNA without overhangs. Data are presented as mean ± s.d. (n = 3 independent experiments).

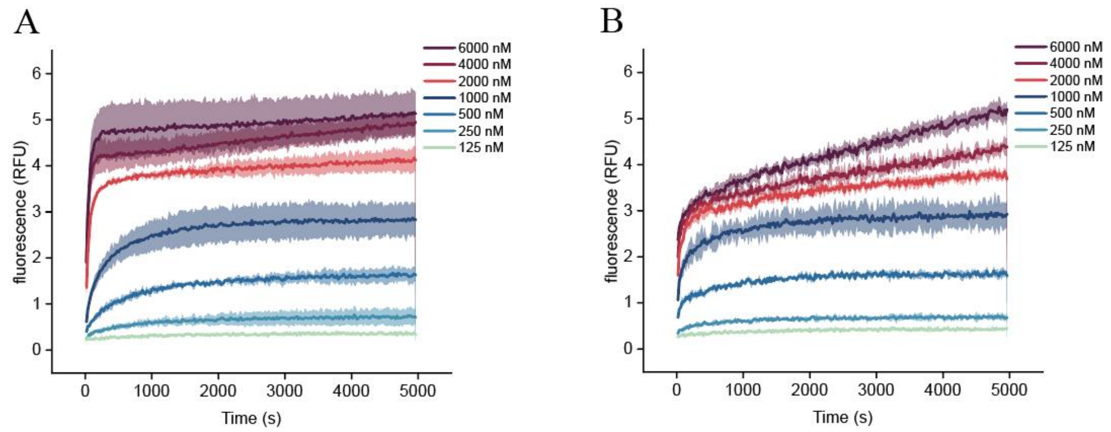


Supplementary Figure S3. *Trans*-cleavage kinetics of LbuCas13a activated by contiguous target and Strategy I. (A) *Trans*-cleavage progress curves for LbuCas13a activated with the contiguous target (T28). (B) *Trans*-cleavage progress curves for LbuCas13a activated with Strategy I (a14_o8 + b14_o8). Data in panels A and B are obtained at a fixed activated enzyme concentration of 1 nM. Data are presented as mean ± s.d. (n = 3 independent experiments).

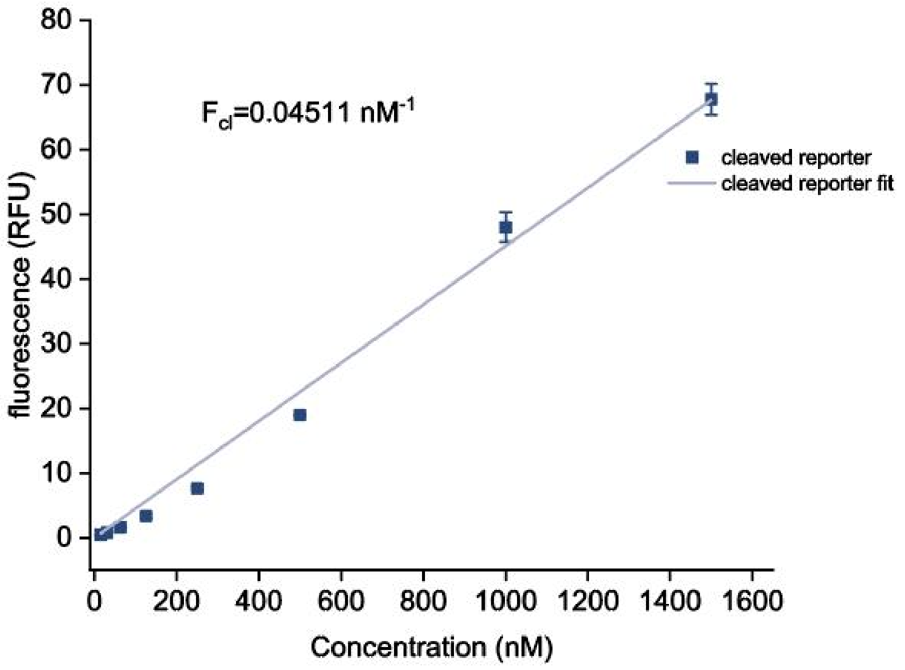


Supplementary Figure S4. Calibration curve of fluorescence intensity versus concentration of the cleaved RNA reporter. Data are presented as mean ± s.d. (n = 3 independent experiments).

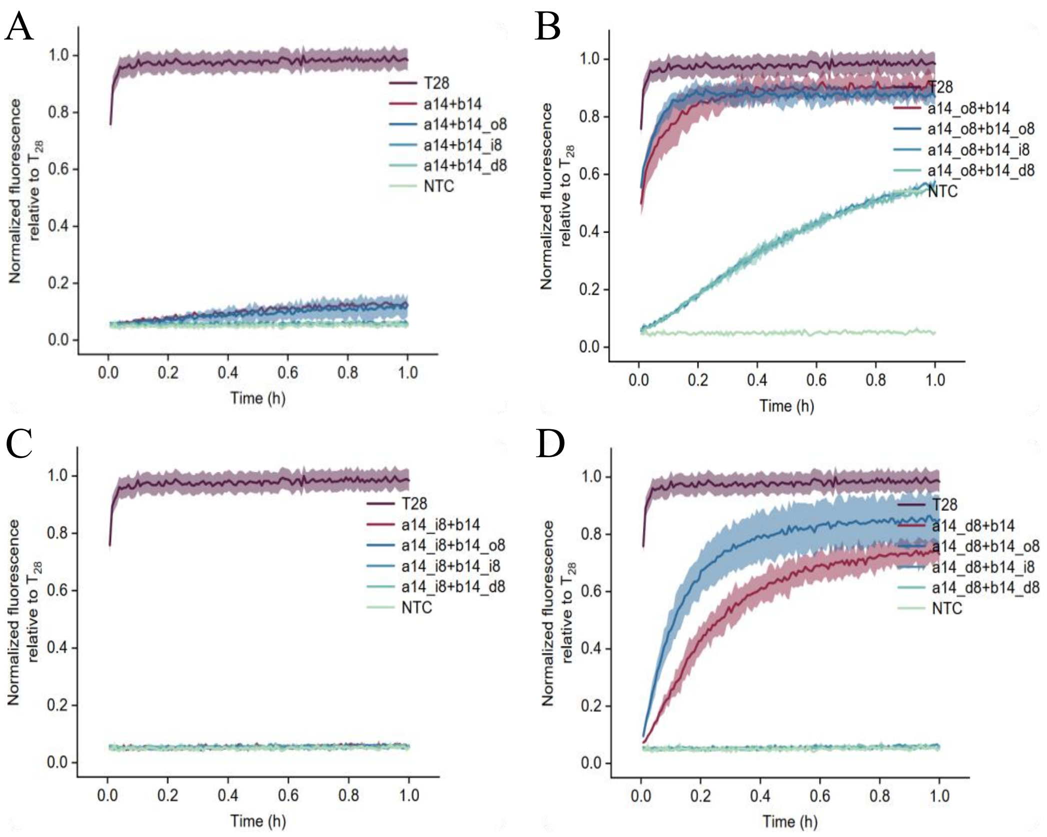


Supplementary Figure S5. LbuCas13a *trans*-cleavage activity mediated by noncontiguous DNA with varying b module overhang statuses (none, outer, inner) under four fixed a module conditions. (A) A module with no overhang. (B) A module with outer overhang. (C) A module with inner overhang. (D) A module with dual overhang. Data are presented as mean ± s.d. (n = 3 independent experiments).

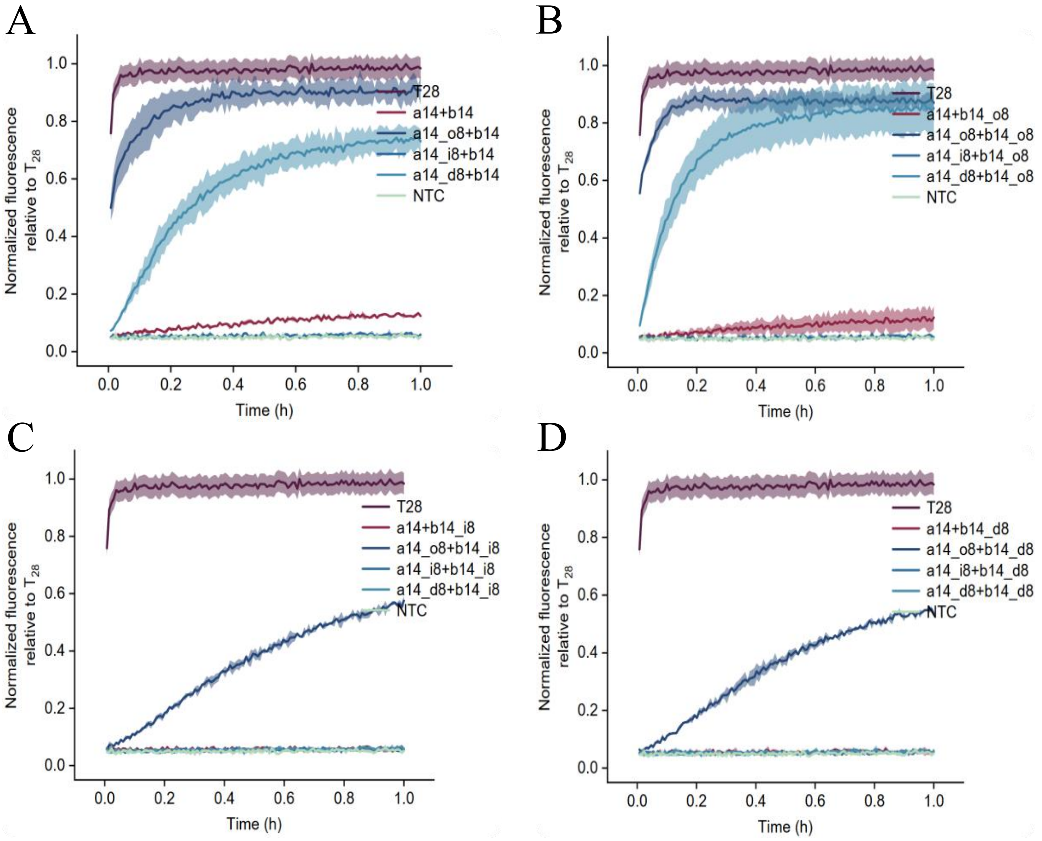


Supplementary Figure S6. LbuCas13a *trans*-cleavage activity mediated by noncontiguous DNA with varying a module overhang statuses (none, outer, inner) under four fixed b module conditions. (A) B module with no overhang. (B) B module with outer overhang. (C) B module with inner overhang. (D) B module with dual overhang. Data are presented as mean ± s.d. (n = 3 independent experiments).

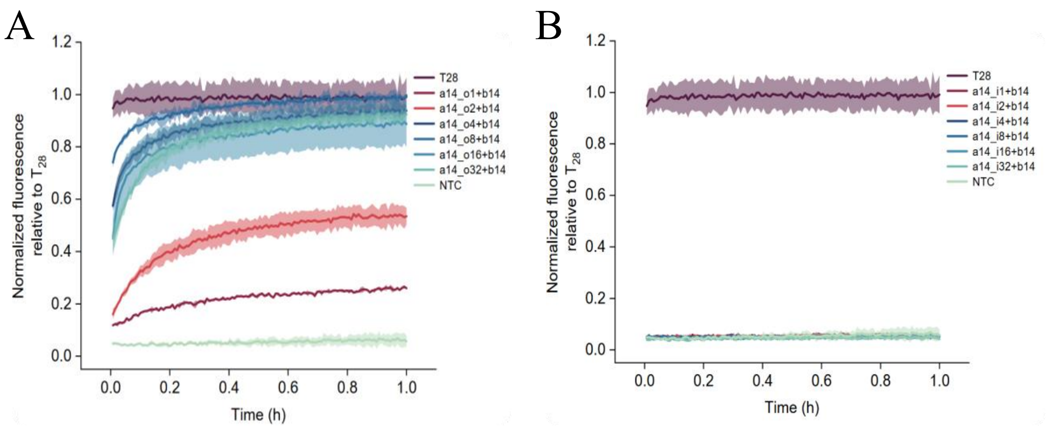


Supplementary Figure S7. Effects of varying a module overhang length on LbuCas13a *trans*-cleavage activity. (A) Fluorescence rates of LbuCas13a activation with increasing lengths of outer overhang in the a module (a14_ox), while the b module is fixed with no overhang (b14). (B) Fluorescence rates of LbuCas13a activation with increasing lengths of inner overhang in the a module (a14_ix), while the b module is fixed with no overhang (b14). Data are presented as mean ± s.d. (n = 3 independent experiments).

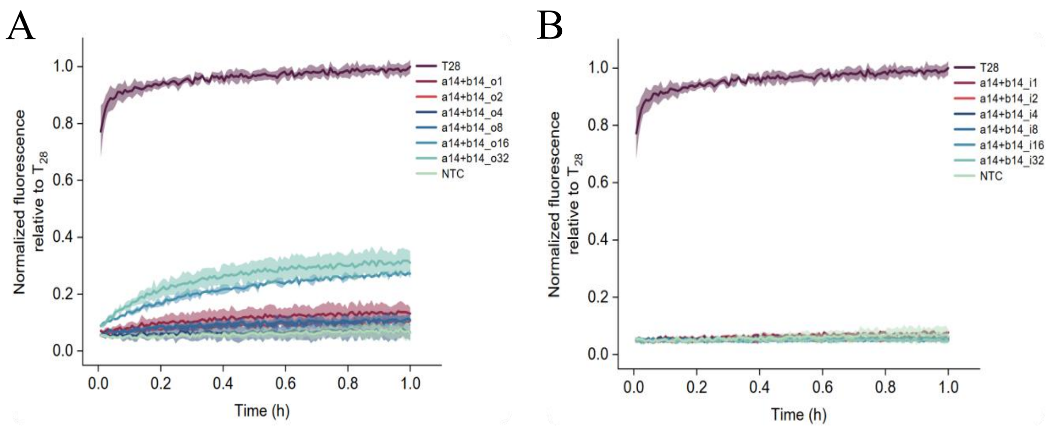


Supplementary Figure S8. Effects of varying b module overhang length on LbuCas13a *trans*-cleavage activity. (A) Fluorescence rates of LbuCas13a activation with increasing lengths of outer overhang in the b module (b14_ox), while the a module is fixed with no overhang (a14). (B) Fluorescence rates of LbuCas13a activation with increasing lengths of inner overhang in the b module (b14_ix), while the a module is fixed with no overhang (a14). Data are presented as mean ± s.d. (n = 3 independent experiments).

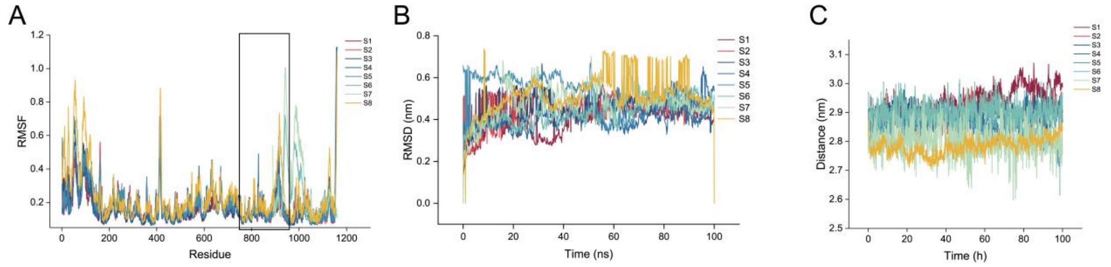


Supplementary Figure S9. Molecular dynamics analysis of LbuCas13a-DNA complexes. (A) Root-mean-square fluctuation (RMSF) of LbuCas13a residues in complexes with S1-S8. (B) Time evolution of backbone root-mean-square deviation (RMSD) in complexes with S1-S8. (C) Time evolution of the center-of-mass distance between the Helical-1 and HEPN2 domains of LbuCas13a in complexes with S1-S8.

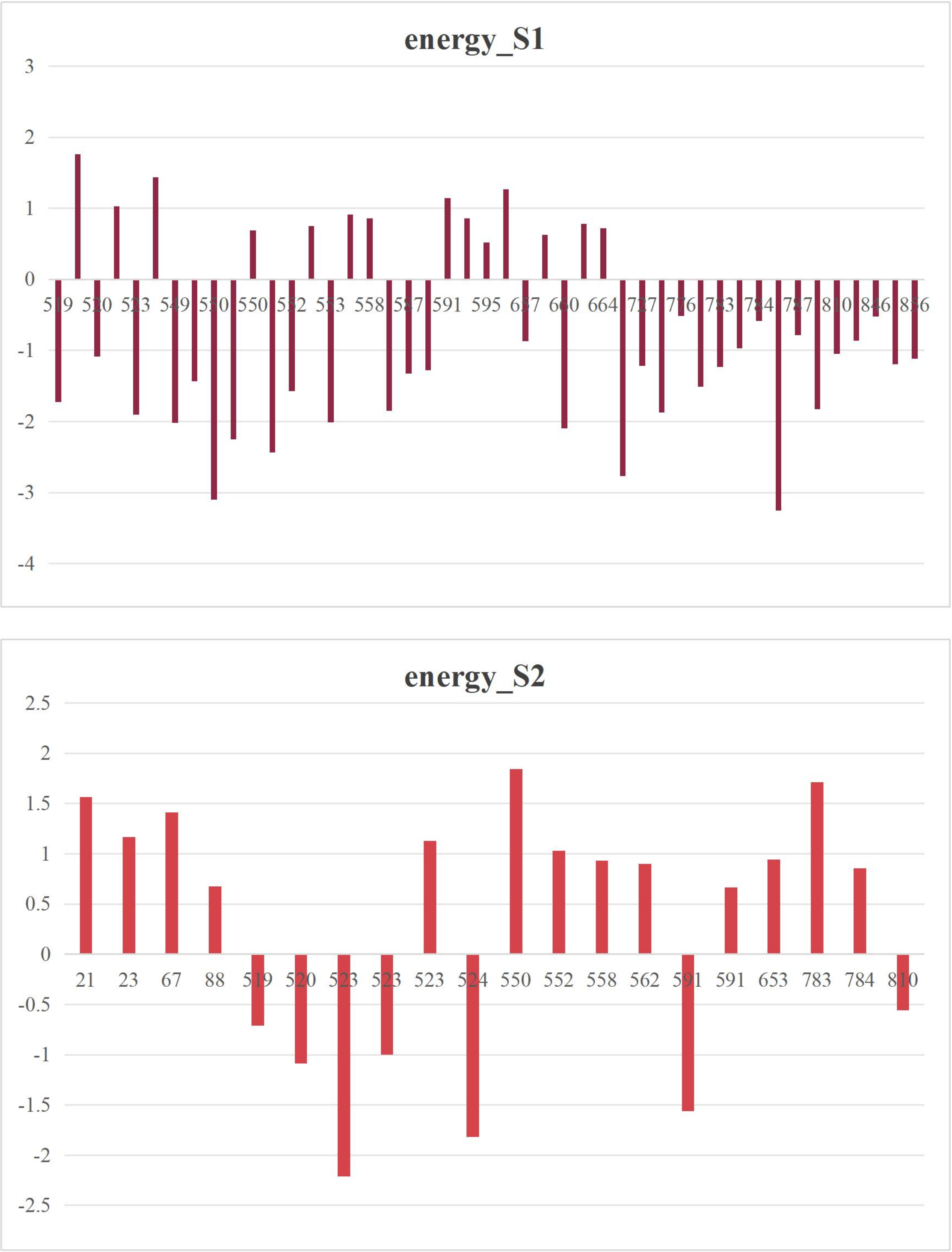


Supplementary Figure S10. Per-residue energy decomposition analysis of LbuCas13a binding to S1 and S2 DNA substrates. Positively charged lysine (Lys) and arginine (Arg) residues serve as the major energetic "hotspots" contributing to LbuCas13a-DNA interactions.

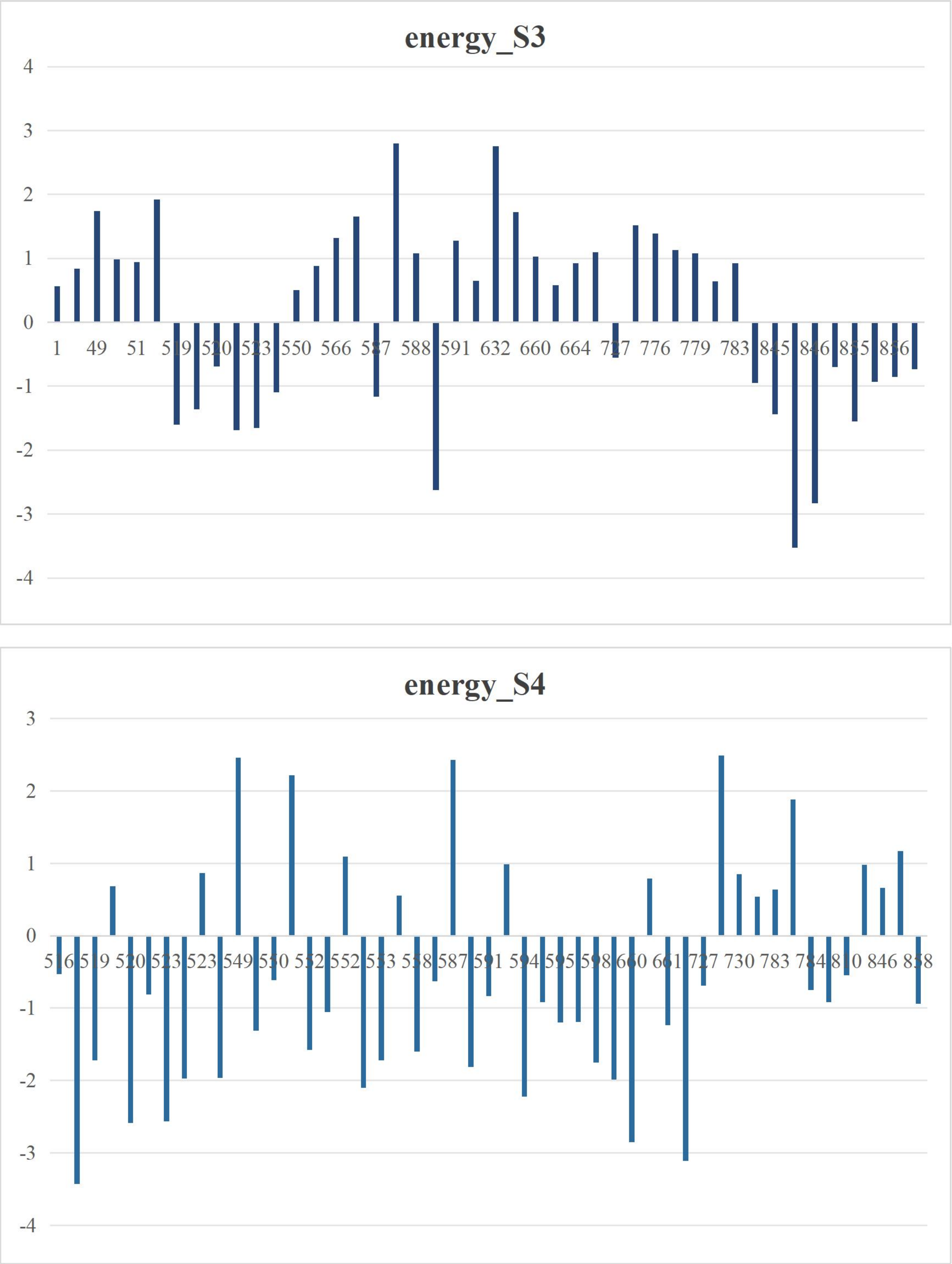


Supplementary Figure S11. Per-residue energy decomposition analysis of LbuCas13a binding to S3 and S4 DNA substrates. Positively charged lysine (Lys) and arginine (Arg) residues serve as the major energetic "hotspots" contributing to LbuCas13a-DNA interactions.

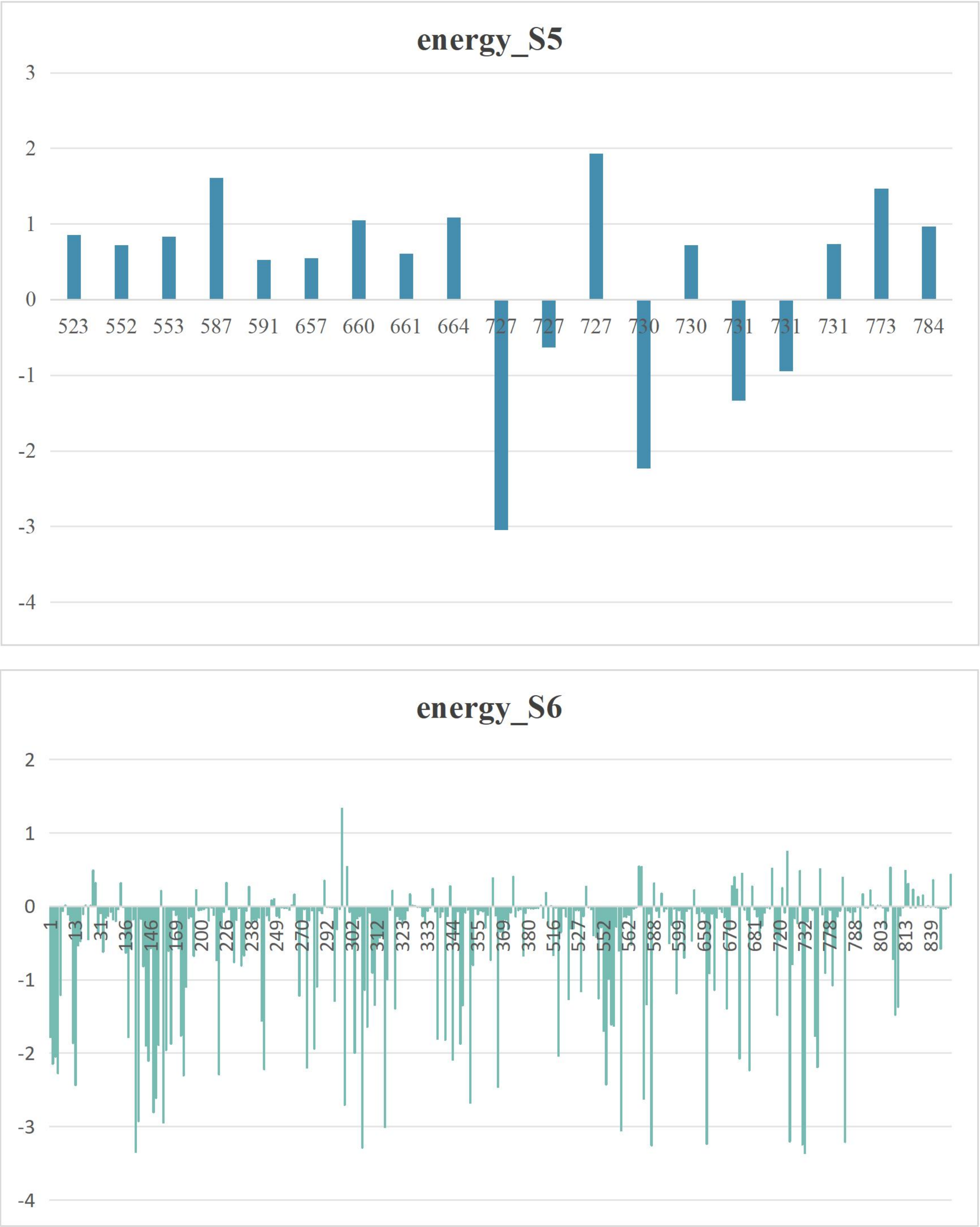


Supplementary Figure S12. Per-residue energy decomposition analysis of LbuCas13a binding to S5 and S6 DNA substrates. Positively charged lysine (Lys) and arginine (Arg) residues serve as the major energetic "hotspots" contributing to LbuCas13a-DNA interactions.

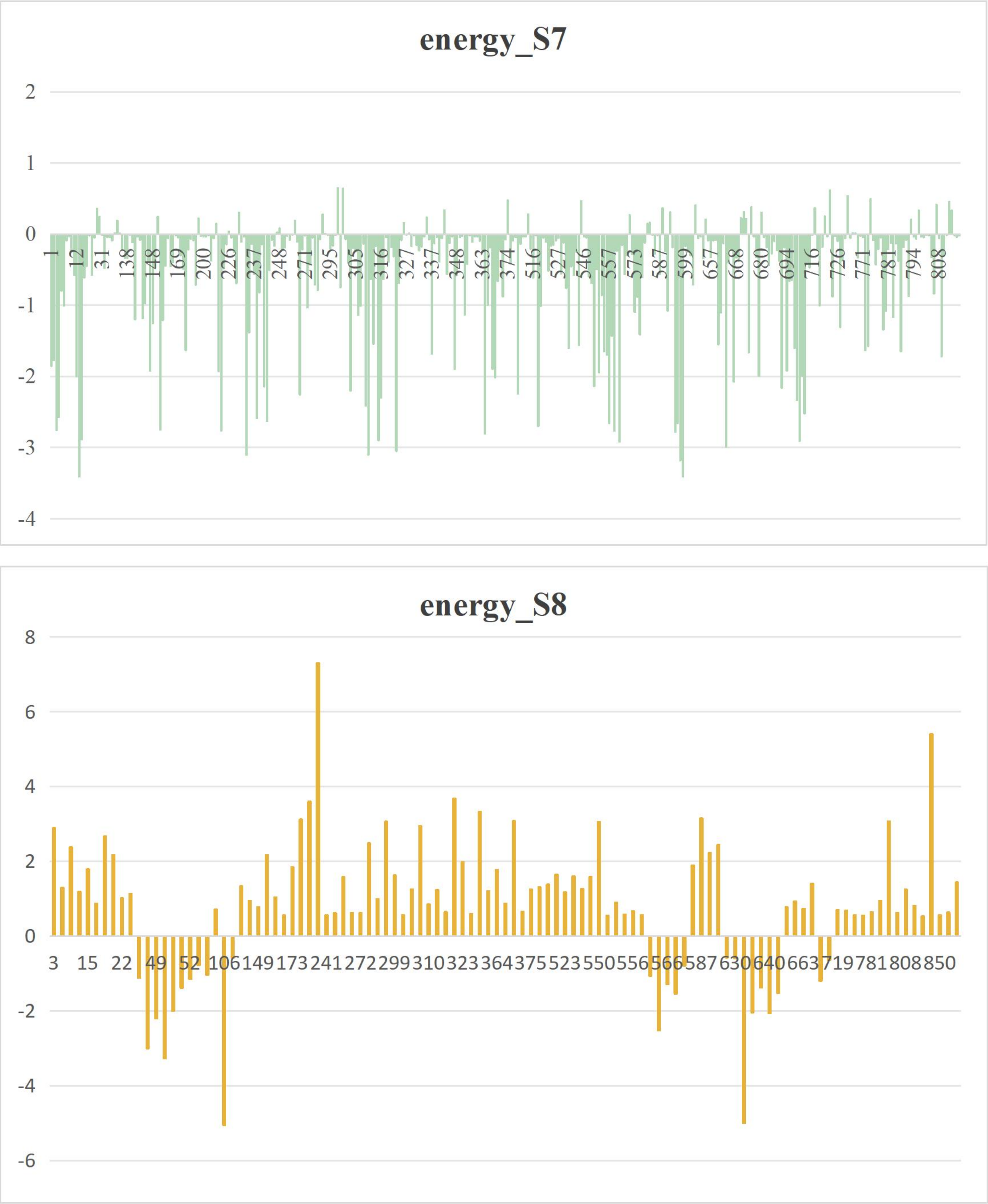


Supplementary Figure S13. Per-residue energy decomposition analysis of LbuCas13a binding to S7 and S8 DNA substrates. Positively charged lysine (Lys) and arginine (Arg) residues serve as the major energetic "hotspots" contributing to LbuCas13a-DNA interactions.

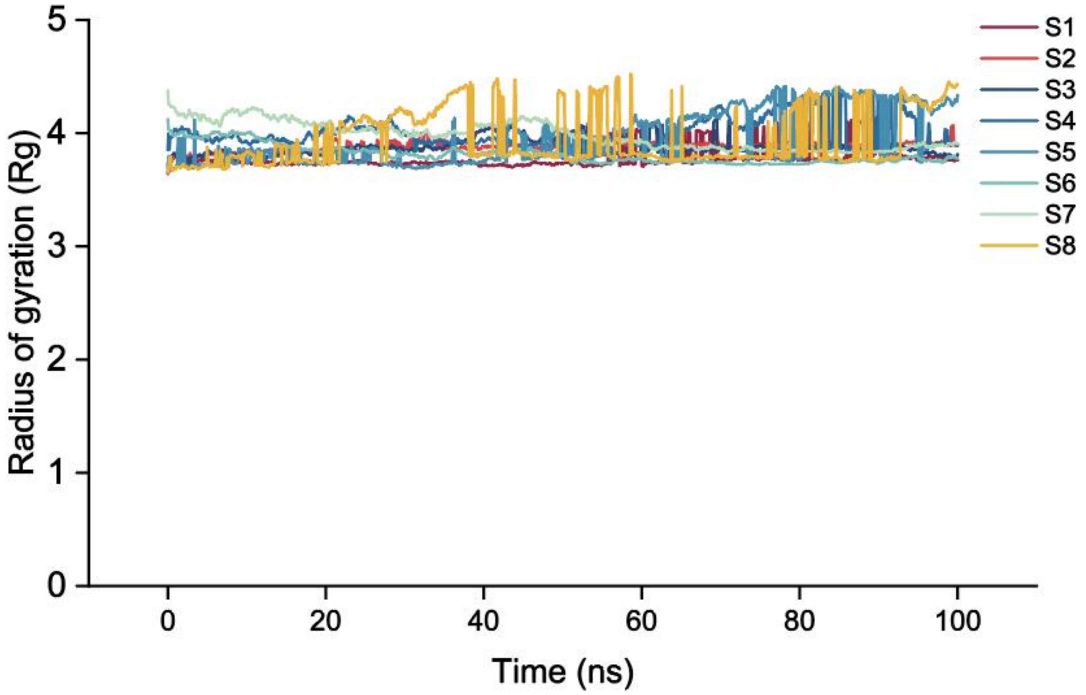

Supplementary Figure S14. Analysis of structural compactness of LbuCas13a upon DNA binding.

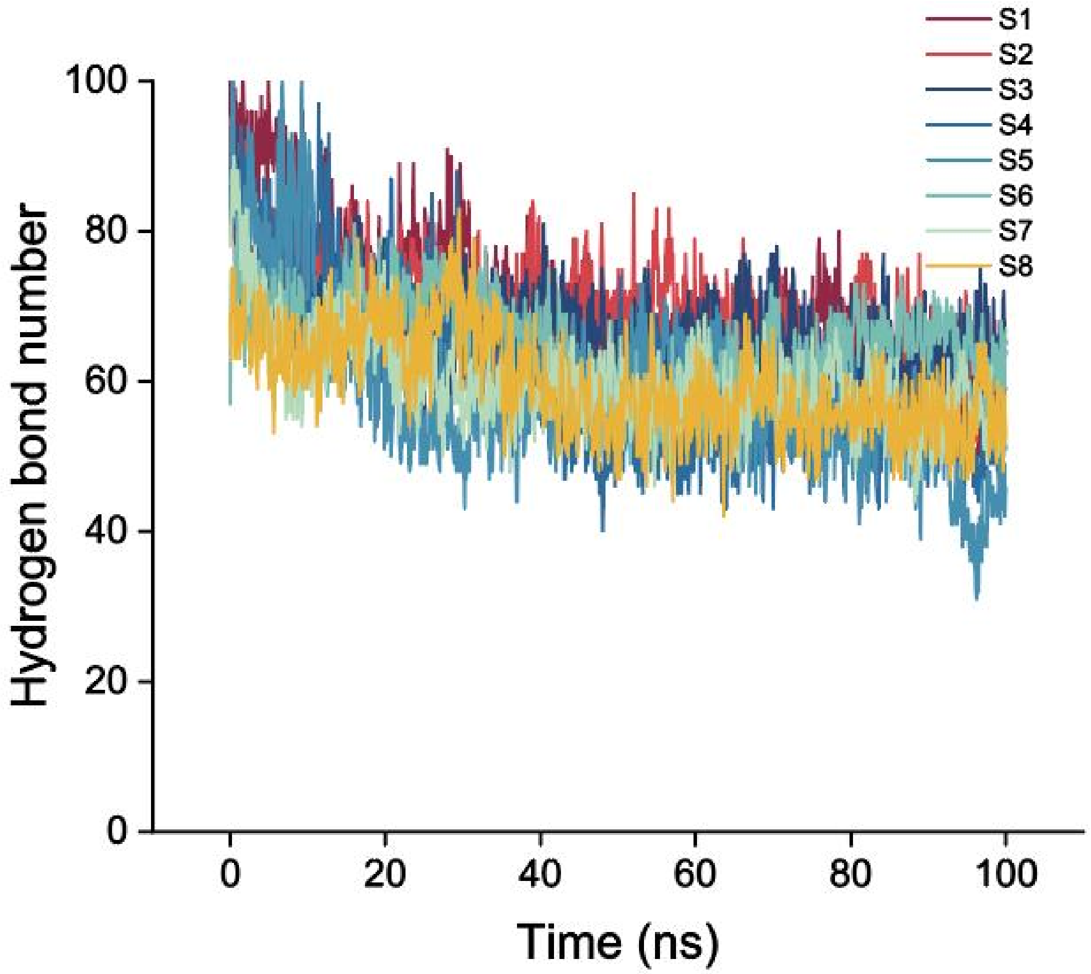


Supplementary Figure S15. Intermolecular hydrogen bond analysis of LbuCas13a-DNA complexes. The time-dependent profiles and average numbers of hydrogen bonds correlate with the binding free energy ranking of different DNA substrates.

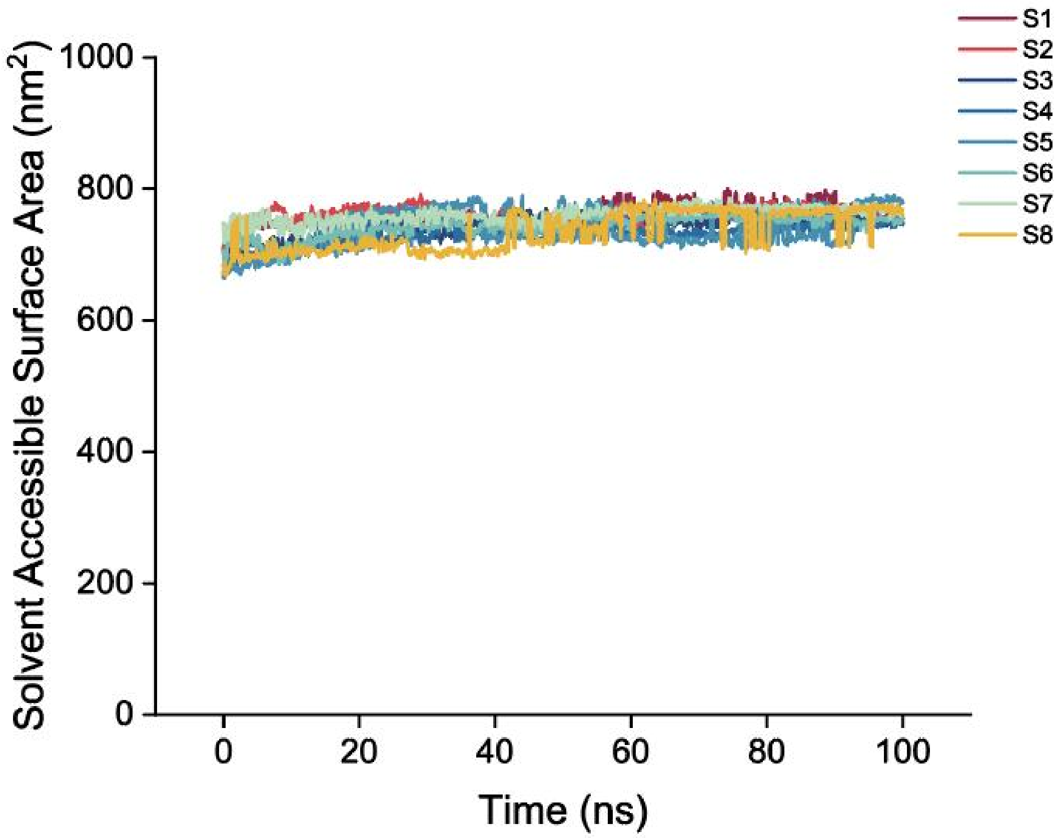


Supplementary Figure S16. Hydrophobic effect analysis of LbuCas13a-DNA complexes revealed by solvent-accessible surface area.

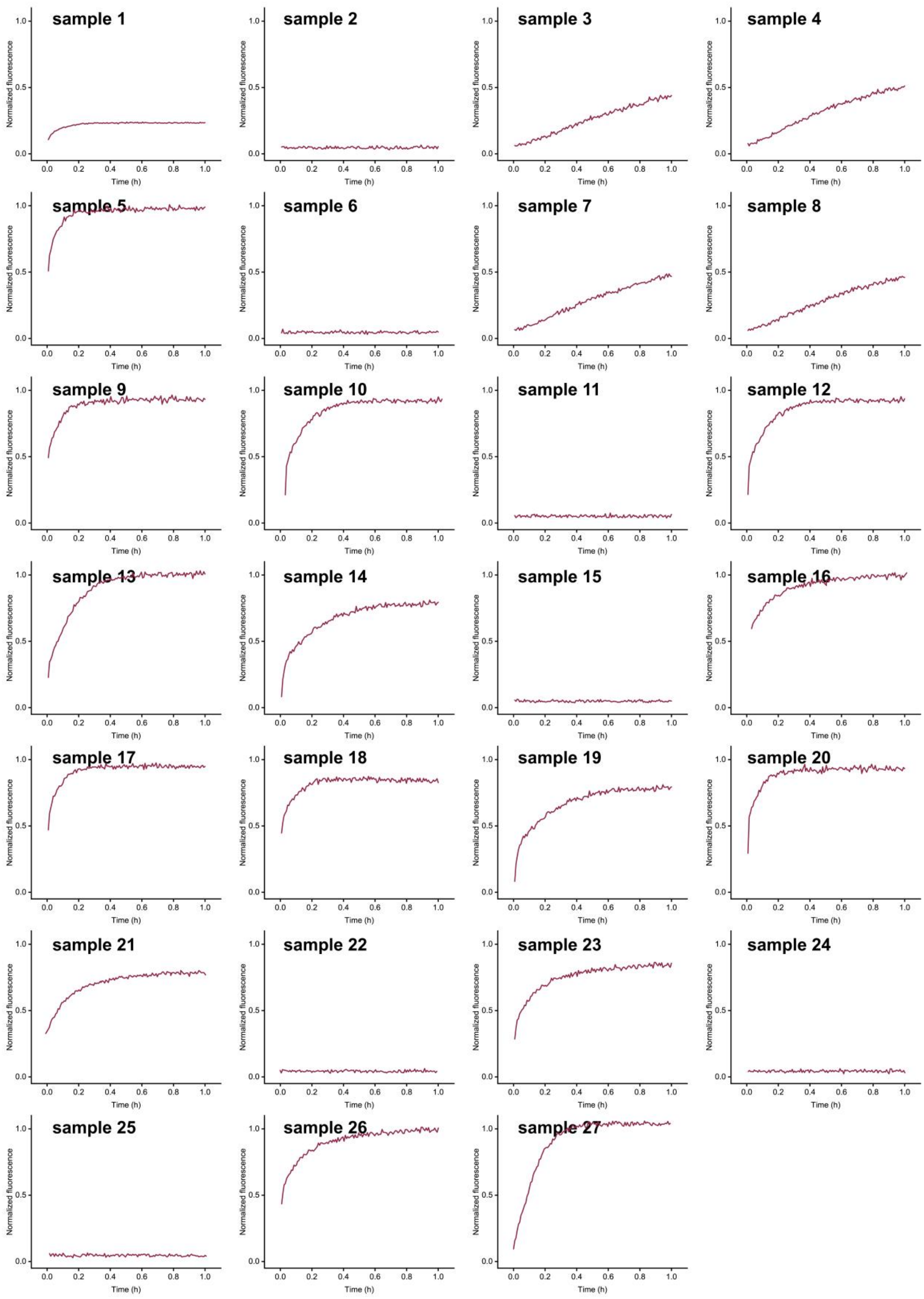


Supplementary Figure S17. Real-time fluorescence curves of the LbuCas13a assay for glioma tissue samples.

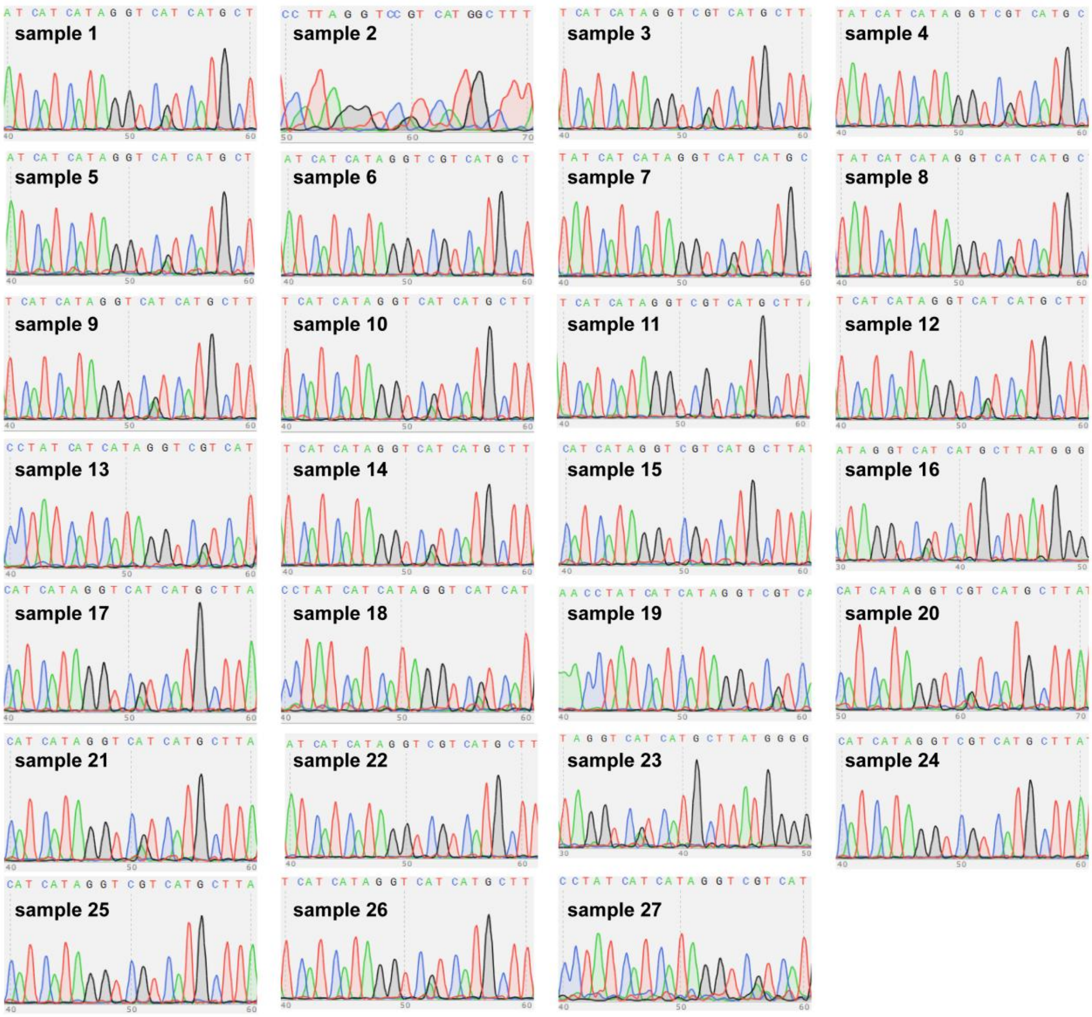


Supplementary Figure S18. Sanger results of brain glioma samples.

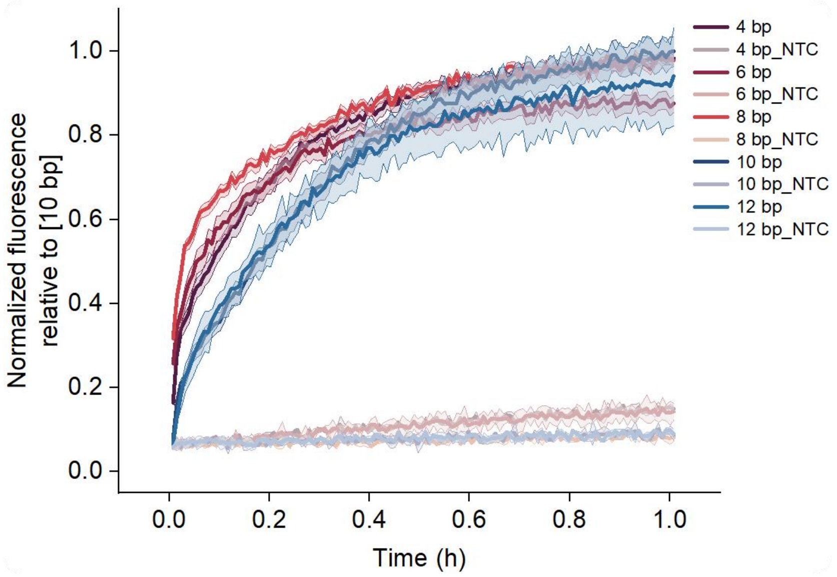


Supplementary Figure S19. Optimization of blocking strand complementary length for APE1-triggered LbuCas13a activation. Data are presented as mean ± s.d. (n = 3 independent experiments).

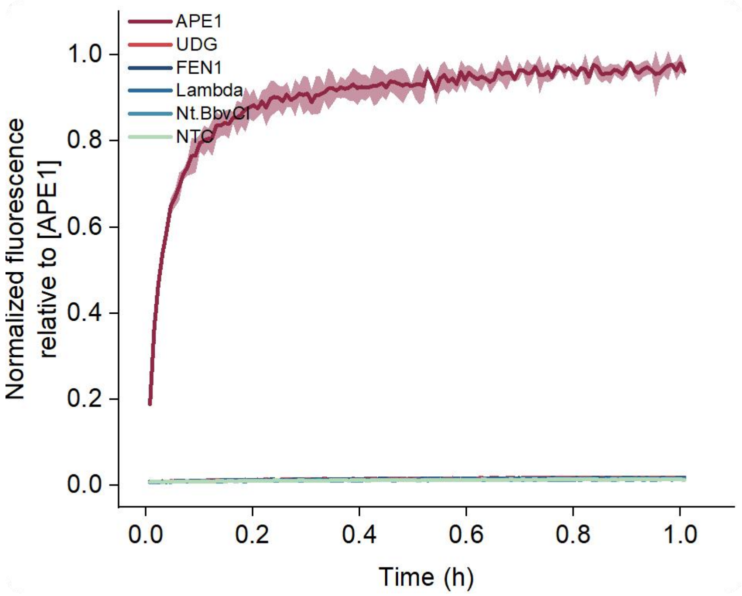


Supplementary Figure S20. Specificity evaluation of the ACROSS platform for APE1 detection. Fluorescence signals generated by the ACROSS system upon incubation with APE1, UDG, FEN1, Lambda exonuclease, and Nt.BbvCI. Data are presented as mean ± s.d. (n = 3 independent experiments).

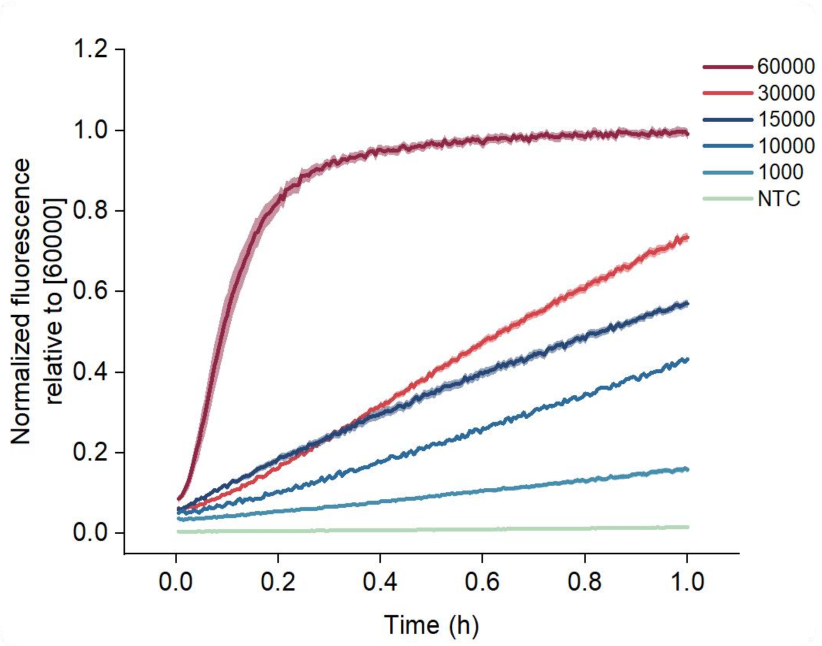


Supplementary Figure S21. Fluorescence response of the ACROSS system to lysates derived from increasing numbers of HeLa cells. Data are presented as mean ± s.d. (n = 3 independent experiments).

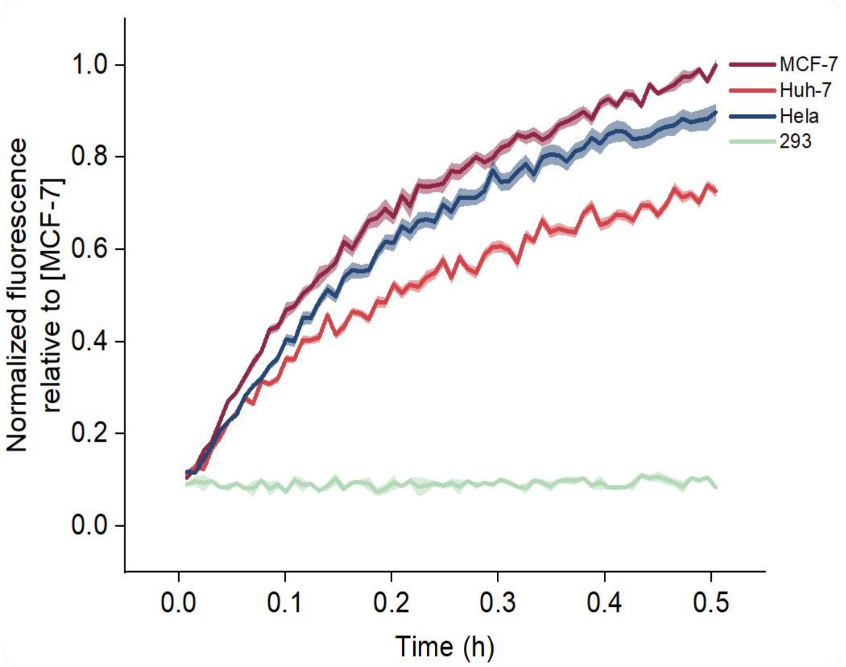


Supplementary Figure S22. Fluorescence response of the ACROSS system to lysates from four human cell lines at equal cell numbers ($1 \times 10^4$ cells). Data are presented as mean ± s.d. (n = 3 independent experiments).

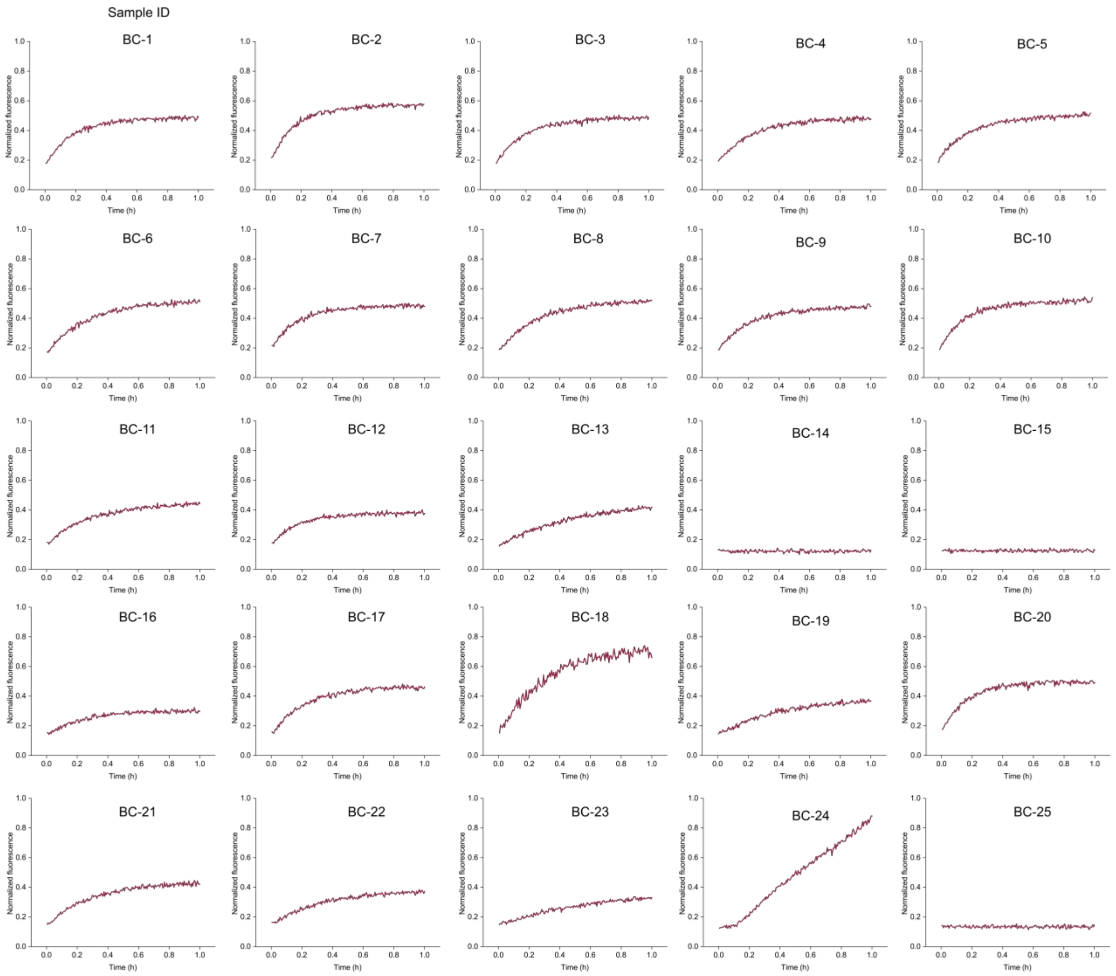


Supplementary Figure S23. Real-time fluorescence kinetics of ACROSS for serum APE1 detection in breast cancer patients (BC 1–25). Fluorescence progress curves of the ACROSS system in response to serum samples from 25 breast cancer patients (BC-1 to BC-25).

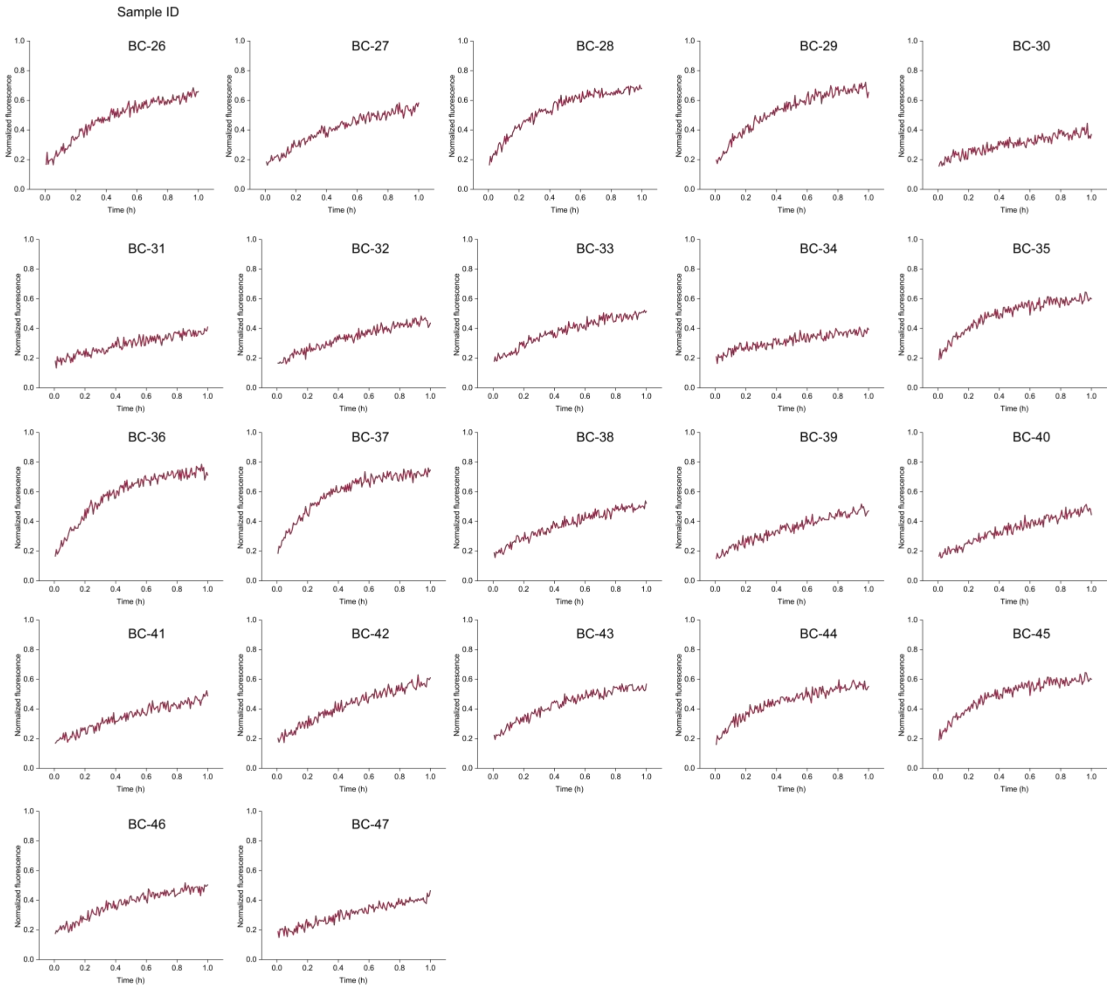


Supplementary Figure S24. Real-time fluorescence kinetics of ACROSS for serum APE1 detection in breast cancer patients (BC 26–47). Fluorescence progress curves of the ACROSS system in response to serum samples from 22 breast cancer patients (BC-26 to BC-47).

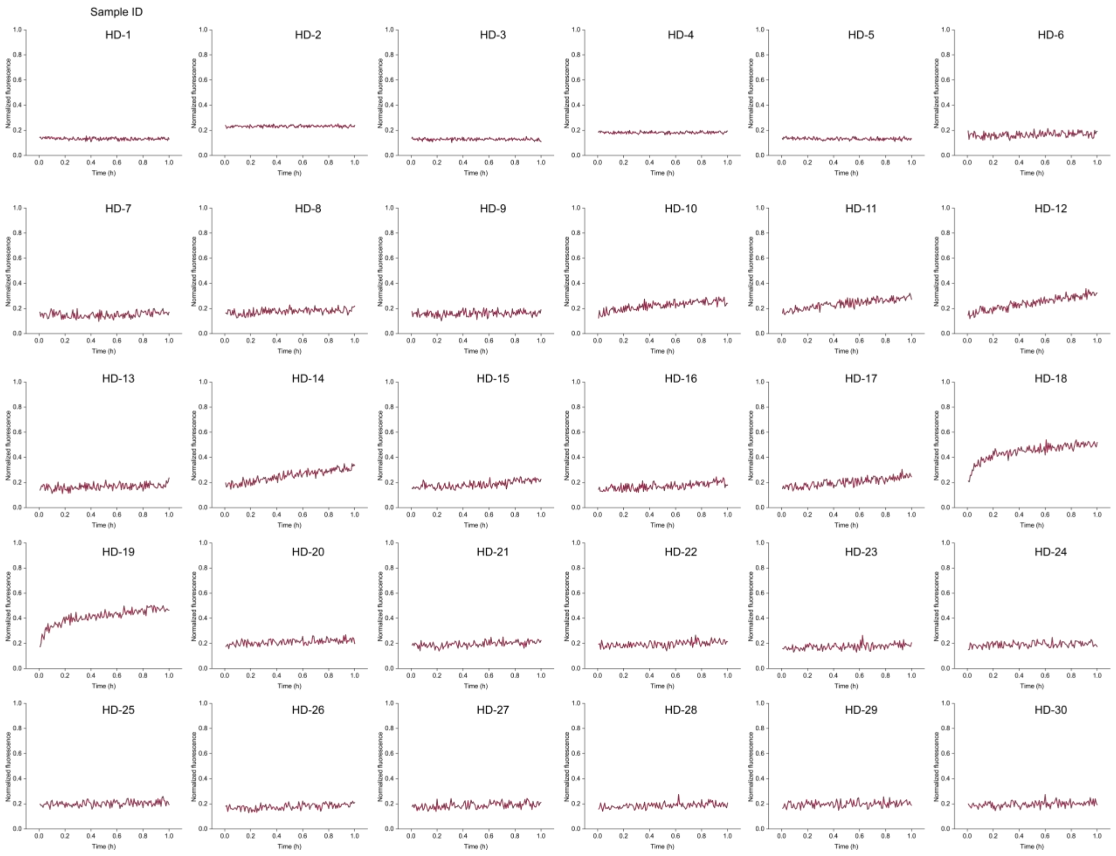


Supplementary Figure S25. Real-time fluorescence kinetics of ACROSS for serum APE1 detection in healthy donors (HD 1–30). Fluorescence progress curves of the ACROSS system in response to serum samples from 30 healthy donors (HD-1 to HD-30).

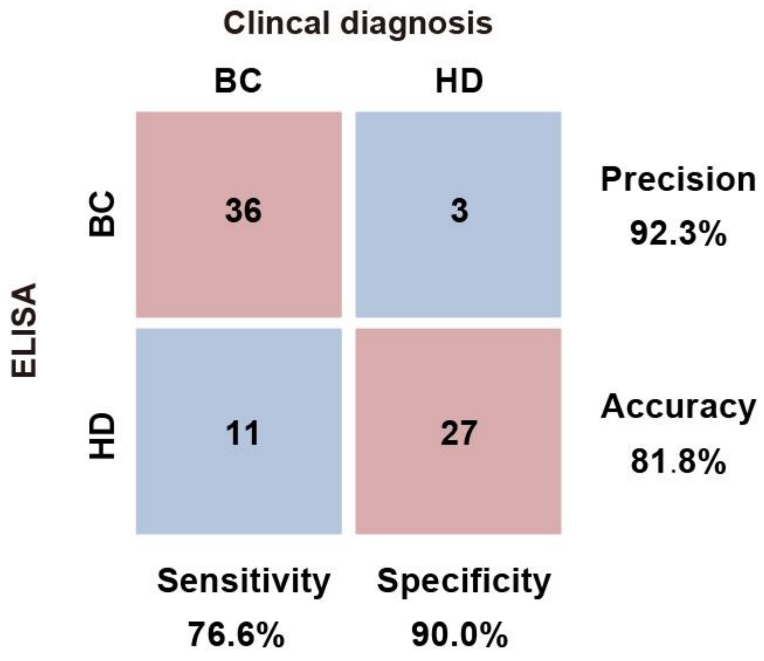


Supplementary Figure S26. Confusion matrix of ELISA for serum APE1 detection in breast cancer patients and healthy donors. Confusion matrix summarizing the diagnostic performance of the commercial ELISA kit for APE1 detection in serum samples from breast cancer patients (n = 47) and healthy donors (n = 30). Using an optimized cutoff value, ELISA demonstrated a sensitivity of 76.6%, specificity of 90.0%, overall accuracy of 81.8%, and positive predictive value (PPV) of 92.3%, as described in the main text.

**Supplementary Table S1. Oligonucleotides used in this work.**

| Name | Sequence 5'-3' |
|---|---|
| crRNA | rGrArCrCrArCrCrCrCrArArArArArUrGrArArGrGrGrGrArCr UrArArArArCrArGrUrUrUrCrUrGrArArGrUrArGrArUrArUr GrGrCrArGrCrArCrArU |
| Target DNA | ATGTGCTGCCATATCTACTTCAGAAACT |
| a22 | TGCCATATCTACTTCAGAAACT |
| a20 | CCATATCTACTTCAGAAACT |
| a18 | ATATCTACTTCAGAAACT |
| a16 | ATCTACTTCAGAAACT |
| a14 | CTACTTCAGAAACT |
| a12 | ACTTCAGAAACT |
| a10 | TTCAGAAACT |
| a8 | CAGAAACT |
| a6 | GAAACT |
| a6 | ATGTGC |
| b8 | ATGTGCTG |
| b10 | ATGTGCTGCC |
| b12 | ATGTGCTGCCAT |
| b14 | ATGTGCTGCCATAT |
| b16 | ATGTGCTGCCATATCT |
| b18 | ATGTGCTGCCATATCTAC |
| b20 | ATGTGCTGCCATATCTACTT |
| b22 | ATGTGCTGCCATATCTACTTCA |
| a22_o8 | TGCCATATCTACTTCAGAAACTTTTTTTTT |
| a20_o8 | CCATATCTACTTCAGAAACTTTTTTTTT |
| a18_o8 | ATATCTACTTCAGAAACTTTTTTTTT |
| a16_o8 | ATCTACTTCAGAAACTTTTTTTTT |
| a14_o8 | CTACTTCAGAAACTTTTTTTTT |
| a12_o8 | ACTTCAGAAACTTTTTTTTT |
| a10_o8 | TTCAGAAACTTTTTTTTT |
| a8_o8 | CAGAAACTTTTTTTTT |
| a6_o8 | GAAACTTTTTTTTT |
| b6_o8 | TTTTTTTTATGTGC |
| b8_o8 | TTTTTTTTATGTGCTG |
| b10_o8 | TTTTTTTTATGTGCTGCC |
| b12_o8 | TTTTTTTTATGTGCTGCCAT |
| b14_o8 | TTTTTTTTATGTGCTGCCATAT |
| b16_o8 | TTTTTTTTATGTGCTGCCATATCT |
| b18_o8 | TTTTTTTTATGTGCTGCCATATCTAC |
| b20_o8 | TTTTTTTTATGTGCTGCCATATCTACTT |
| b22_o8 | TTTTTTTTATGTGCTGCCATATCTACTTCA |
| a22_i8 | TTTTTTTTTGCCATATCTACTTCAGAAACT |

| | |
|---|---|
| a20_i8 | TTTTTTTTCCATATCTACTTCAGAAACT |
| a18_i8 | TTTTTTTTATATCTACTTCAGAAACT |
| a16_i8 | TTTTTTTTATCTACTTCAGAAACT |
| a14_i8 | TTTTTTTTCTACTTCAGAAACT |
| a12_i8 | TTTTTTTTACTTCAGAAACT |
| a10_i8 | TTTTTTTTTTCAGAAACT |
| a8_i8 | TTTTTTTTCAGAAACT |
| a6_i8 | TTTTTTTTGAAACT |
| b6_i8 | ATGTGCTTTTTTTT |
| b8_i8 | ATGTGCTGTTTTTTTT |
| b10_i8 | ATGTGCTGCCTTTTTTTT |
| b12_i8 | ATGTGCTGCCATTTTTTTTT |
| b14_i8 | ATGTGCTGCCATATTTTTTTTT |
| b16_i8 | ATGTGCTGCCATATCTTTTTTTTT |
| b18_i8 | ATGTGCTGCCATATCTACTTTTTTTT |
| b20_i8 | ATGTGCTGCCATATCTACTTTTTTTTTT |
| b22_i8 | ATGTGCTGCCATATCTACTTCATTTTTTTT |
| P-AP-a14-o8 | TAGCATTAAGTTGTAAXCTACTTCAGAAACTTTTTTTTT |
| P1_4 | GTAGATTACAACTTAATGCTA |
| P1_6 | AAGTAGATTACAACTTAATGCTA |
| P1_8 | TGAAGTAGATTACAACTTAATGCTA |
| P1_10 | TCTGAAGTAGATTACAACTTAATGCTA |
| P1_12 | TTTCTGAAGTAGATTACAACTTAATGCTA |
| Random RNA | UUUUUGGGGGUUUUUGGGGGUUUUUGGG |
| Overhang-T | TTTTTTTTATGTGCTGCCATAT‘+’CTACTTCAGAAACTT TTTTTTT |
| Overhang-A | AAAAAAAAATGTGCTGCCATAT‘+’CTACTTCAGAAAC TAAAAAAAA |
| Overhang-C | CCCCCCCCATGTGCTGCCATAT‘+’CTACTTCAGAAACT CCCCCCCC |
| Overhang-G | GGGGGGGGATGTGCTGCCATAT‘+’CTACTTCAGAAAC TGGGGGGGG |
| a14_d8 | TTTTTTTTCTACTTCAGAAACTTTTTTTTT |
| b14_d8 | TTTTTTTTATGTGCTGCCATATTTTTTTTT |
| a14_o1 | CTACTTCAGAAACTT |
| a14_o2 | CTACTTCAGAAACTTT |
| a14_o4 | CTACTTCAGAAACTTTTT |
| a14_o8 | CTACTTCAGAAACTTTTTTTTT |
| a14_o16 | CTACTTCAGAAACTTTTTTTTTTTTTTTTT |
| a14_o32 | CTACTTCAGAAACTTTTTTTTTTTTTTTTTTTTTTTTTTT TTTTTT |
| a14_i1 | TCTACTTCAGAAACT |
| a14_i2 | TTCTACTTCAGAAACT |
| a14_i4 | TTTTCTACTTCAGAAACT |

| | |
|---|---|
| a14_i8 | TTTTTTTTCTACTTCAGAAACT |
| a14_i16 | TTTTTTTTTTTTTTTTCTACTTCAGAAACT |
| a14_i32 | TTTTTTTTTTTTTTTTTTTTTTTTTTTTTTTTCTACTTCAGAAACT |
| b14_o1 | TATGTGCTGCCATAT |
| b14_o2 | TTATGTGCTGCCATAT |
| b14_o4 | TTTTATGTGCTGCCATAT |
| b14_o8 | TTTTTTTTATGTGCTGCCATAT |
| b14_o16 | TTTTTTTTTTTTTTTTATGTGCTGCCATAT |
| b14_o32 | TTTTTTTTTTTTTTTTTTTTTTTTTTTTTTTTATGTGCTGCCATAT |
| b14_i1 | ATGTGCTGCCATATT |
| b14_i2 | ATGTGCTGCCATATTT |
| b14_i4 | ATGTGCTGCCATATTTTT |
| b14_i8 | ATGTGCTGCCATATTTTTTTTT |
| b14_i16 | ATGTGCTGCCATATTTTTTTTTTTTTTTTT |
| b14_i32 | ATGTGCTGCCATATTTTTTTTTTTTTTTTTTTTTTTTTTTTTTTTT |
| RNA reporter | HEX-UUUUU-BHQ2 |
| CrRNA-IDH1 | rGrArCrCrArCrCrCrCrArArArArArUrGrArAGrGrGrGrArCrUrArArArArCrArGrCrArUrGrArUrGrArCrCrUrArUrUrGrCrCrArCrGrArCrUrUrArG |

**Supplementary Table S2. Binding free energy components for LbuCas13a-DNA complexes.**

| ligand | $\Delta G_{VDWALS}$ | $\Delta G_{EEL}$ | $\Delta G_{EBG}$ | $\Delta G_{ESURF}$ | $\Delta G_{GGAS}$ | $\Delta G_{GSOLV}$ | $\Delta G_{TOTAL}$ |
|---|---|---|---|---|---|---|---|
| S1 | -181.62 | -1351.34 | 1379.93 | -21.51 | -1532.95 | 1358.43 | -174.53 |
| S2 | -134.01 | -995.74 | 989.72 | -16.61 | -1129.75 | 973.12 | -156.64 |
| S3 | -109.58 | -835.85 | 806.63 | -13.88 | -945.44 | 792.74 | -152.69 |
| S4 | -99.05 | -1114.35 | 1113.76 | -13.25 | -1213.41 | 1100.51 | -112.90 |
| S5 | -110.79 | -841.05 | 854.70 | -13.88 | -951.84 | 840.82 | -111.02 |
| S6 | -111.70 | -844.14 | 860.27 | -13.36 | -955.84 | 846.91 | -108.93 |
| S7 | -113.39 | -858.49 | 878.53 | -13.80 | -971.88 | 864.73 | -107.15 |
| S8 | -66.08 | -725.51 | 719.00 | -8.75 | -791.59 | 710.25 | -81.34 |

**Supplementary Table S3. Clinical information for IDH1.**

| Sample | Age (years) | Sex | Disease |
|---|---|---|---|
| Patient 1 | 53 | Women | oligodendrogliomas |
| Patient 2 | 50 | Man | glioblastoma |
| Patient 3 | 71 | Women | astrocytomas |
| Patient 4 | 41 | Man | oligodendrogliomas |
| Patient 5 | 43 | Women | astrocytomas |
| Patient 6 | 53 | Man | glioblastoma |
| Patient 7 | 47 | Women | oligodendrogliomas |
| Patient 8 | 61 | Man | astrocytomas |
| Patient 9 | 74 | Women | oligodendrogliomas |
| Patient 10 | 42 | Man | astrocytomas |
| Patient 11 | 38 | Women | glioblastoma |
| Patient 12 | 59 | Man | oligodendrogliomas |
| Patient 13 | 60 | Women | astrocytomas |
| Patient 14 | 72 | Man | oligodendrogliomas |
| Patient 15 | 55 | Women | glioblastoma |
| Patient 16 | 31 | Man | astrocytomas |
| Patient 17 | 52 | Women | oligodendrogliomas |
| Patient 18 | 76 | Man | astrocytomas |
| Patient 19 | 61 | Women | astrocytomas |
| Patient 20 | 49 | Man | oligodendrogliomas |
| Patient 21 | 66 | Women | astrocytomas |
| Patient 22 | 67 | Man | oligodendrogliomas |
| Patient 23 | 58 | Women | astrocytomas |
| Patient 24 | 52 | Man | glioblastoma |
| Patient 25 | 64 | Women | glioblastoma |
| Patient 26 | 38 | Man | astrocytomas |
| Patient 27 | 49 | Women | astrocytomas |

**Supplementary Table S4. Comparison of the ACROSS platform with other APE1 detection methods.**

| Method | LOD (U/mL) | Linear range (U/mL) | Reference |
|---|---|---|---|
| ACROSS | $1.00\times10^{-5}$ | $1.0\times10^{-5}$ - $1.0\times10^{-1}$ | This work |
| DEP-Cas-APE | $7.66\times10^{-5}$ | $1.0\times10^{-7}$ - $1.0\times10^{-5}$ | 1 |
| EDC | $4.80\times10^{-5}$ | $5.0\times10^{-5}$ - $1.0\times10^{-1}$ | 2 |
| RCRE | $8.86\times10^{-4}$ | $2.0\times10^{-3}$ - $2.0\times10^{-1}$ | 3 |
| ARC | $1.74\times10^{-3}$ | $2.5\times10^{-3}$ - $2.5\times10^{-1}$ | 4 |
| Split activator-based | $1.00\times10^{-5}$ | / | 5 |

**Supplementary Table S5. Recovery results of spiked analytes in complex matrix.**

| Sample | Added (U/mL) | Found (U/mL) | Recovery (%) |
|---|---|---|---|
| 1 | 1 | 1.04 | 104% |
| 2 | $1\times10^{-2}$ | $1.1\times10^{-2}$ | 110% |
| 3 | $1\times10^{-5}$ | $0.97\times10^{-5}$ | 97% |

*These analytes were diluted in 10% serum samples with different concentrations.

**Supplementary Table S6. Clinical information for healthy donors (HD) and breast cancer patients.**

| Sample | Age (years) | ER | PR | Her2 | KI-67(%) | TNM | Stage number | CA15-3 U/mL |
|---|---|---|---|---|---|---|---|---|
| Patient 1 | 22 | + | + | 1+ | + | T2N0M0 | II | 7.5 |
| Patient 2 | 58 | + | + | - | + | T2N0M0 | II | 8.4 |
| Patient 3 | 60 | 1+ | - | 3+ | + | T2N1M0 | II | 9.2 |
| Patient 4 | 40 | + | + | 1+ | + | T2N2M0 | III | 4.8 |
| Patient 5 | 66 | - | - | 2+ | + | T2N0M0 | II | 7.0 |
| Patient 6 | 64 | + | 2+ | 2+ | + | T2N1M0 | I | 9.9 |
| Patient 7 | 71 | + | + | 2+ | + | T1N2M0 | III | 17.6 |
| Patient 8 | 47 | + | + | + | + | T1N0M0 | I | 15.7 |
| Patient 9 | 64 | - | - | - | + | T1N0M0 | I | 20.0 |
| Patient 10 | 50 | - | - | 2+ | + | T1N0M0 | I | 12.3 |
| Patient 11 | 55 | 2+ | 1+ | - | + | T2N0M0 | II | 7.6 |
| Patient 12 | 45 | + | + | 3+ | + | T2N0M0 | II | 5.5 |
| Patient 13 | 40 | - | - | 3+ | + | T3N3M0 | III | 7.4 |
| Patient 14 | 40 | 3+ | 3+ | - | + | T2N1M0 | II | 13.5 |
| Patient 15 | 59 | + | - | 2+ | + | T2N0M0 | II | 8.8 |
| Patient 16 | 71 | + | + | 1+ | + | T1N0M0 | I | 5.4 |
| Patient 17 | 58 | - | - | 3+ | + | T2N2M0 | III | 21.6 |
| Patient 18 | 53 | - | - | - | + | T1N0M0 | I | 12.0 |
| Patient 19 | 51 | 3+ | 2+ | 2+ | + | T1N0M0 | I | 6.4 |
| Patient 20 | 55 | - | - | 3+ | + | T2N0M0 | II | 4.9 |
| Patient 21 | 54 | + | + | - | + | T2N1M0 | II | 9.4 |
| Patient 22 | 62 | + | - | 1+ | + | T2N0M0 | II | 4.0 |
| Patient 23 | 48 | + | + | 2+ | + | T2N1M0 | II | 12.8 |
| Patient 24 | 55 | - | - | 3+ | + | T3N0M0 | III | 23.8 |
| Patient 25 | 61 | + | + | - | + | T3N3M0 | III | 14.1 |
| Patient 26 | 51 | - | - | 3+ | + | T1N0M0 | I | 22.1 |
| Patient 27 | 52 | - | - | 3+ | + | T3N3M0 | III | 3.3 |
| Patient 28 | 60 | - | - | 3+ | + | T3N3M0 | III | 35.3 |
| Patient 29 | 57 | + | - | - | + | T1N0M0 | I | 13.9 |
| Patient 30 | 50 | - | - | - | + | T2N1M0 | II | 6.3 |
| Patient 31 | 52 | - | - | 3+ | + | T2N0M0 | II | 12.3 |
| Patient 32 | 79 | 3+ | 3+ | 1+ | + | T2N1M0 | II | 10.6 |
| Patient 33 | 53 | 3+ | 3+ | - | + | T2N0M0 | II | 8.3 |
| Patient 34 | 69 | - | + | 2+ | + | T3N2M0 | III | 12.8 |
| Patient 35 | 73 | - | - | 1+ | + | T1N0M0 | I | 8.9 |
| Patient 36 | 62 | 3+ | - | 1+ | + | T2N0M0 | II | 3.4 |
| Patient 37 | 46 | + | + | 1+ | + | T1N0M0 | I | 13.0 |
| Patient 38 | 40 | - | - | 2+ | + | T1N0M0 | I | 13.1 |
| Patient 39 | 72 | + | + | - | + | T3N2M0 | III | 18.2 |

| | | | | | | | | |
|---|---|---|---|---|---|---|---|---|
| Patient 40 | 41 | + | - | 2+ | + | T3N2M0 | III | 30.5 |
| Patient 41 | 49 | + | + | 1+ | + | T1N0M0 | I | 14.8 |
| Patient 42 | 49 | 3+ | 3+ | 2+ | + | T1N0M0 | I | 16.1 |
| Patient 43 | 52 | + | + | - | + | T1N0M0 | I | 4.8 |
| Patient 44 | 45 | + | + | 3+ | + | T2N1M0 | II | 5.2 |
| Patient 45 | 50 | + | + | - | + | T1N0M0 | I | 6.3 |
| Patient 46 | 72 | - | - | 3+ | + | T2N0M0 | I | 11.2 |
| Patient 47 | 40 | - | - | + | + | T2N2M0 | II | 18.3 |
| HD 1 | - | - | - | - | - | - | - | 8.7 |
| HD 2 | - | - | - | - | - | - | - | 8.1 |
| HD 3 | - | - | - | - | - | - | - | 7.2 |
| HD 4 | - | - | - | - | - | - | - | 11.6 |
| HD 5 | - | - | - | - | - | - | - | 5.8 |
| HD 6 | - | - | - | - | - | - | - | 9.3 |
| HD 7 | - | - | - | - | - | - | - | 12.4 |
| HD 8 | - | - | - | - | - | - | - | 6.5 |
| HD 9 | - | - | - | - | - | - | - | 14.9 |
| HD 10 | - | - | - | - | - | - | - | 8.1 |
| HD 11 | - | - | - | - | - | - | - | 10.7 |
| HD 12 | - | - | - | - | - | - | - | 13.2 |
| HD 13 | - | - | - | - | - | - | - | 7.8 |
| HD 14 | - | - | - | - | - | - | - | 15.5 |
| HD 15 | - | - | - | - | - | - | - | 6.2 |
| HD 16 | - | - | - | - | - | - | - | 12.8 |
| HD 17 | - | - | - | - | - | - | - | 9.6 |
| HD 18 | - | - | - | - | - | - | - | 16.1 |
| HD 19 | - | - | - | - | - | - | - | 5.5 |
| HD 20 | - | - | - | - | - | - | - | 10.3 |
| HD 21 | - | - | - | - | - | - | - | 13.7 |
| HD 22 | - | - | - | - | - | - | - | 7 |
| HD 23 | - | - | - | - | - | - | - | 14.4 |
| HD 24 | - | - | - | - | - | - | - | 8.9 |
| HD 25 | - | - | - | - | - | - | - | 17.3 |
| HD 26 | - | - | - | - | - | - | - | 9.2 |
| HD 27 | - | - | - | - | - | - | - | 12 |
| HD 28 | − | − | − | − | − | − | − | 11.1 |
| HD 29 | − | − | − | − | − | − | − | 13.8 |
| HD 30 | − | − | − | − | − | − | − | 10 |

Note: The reference ranges for serum tumor markers are as follows: CEA: 0–5.2 ng/mL; CA15-3: 0–25 U/mL.

Abbreviations: CEA, carcinoembryonic antigen; CA15-3, cancer antigen 15-3.

**References**

1. Song Y, Long J, Wang H et al. High-efficiency detection of APE1 using a defective PAM-driven CRISPR-Cas12a self-catalytic biosensor. Biosens Bioelectron. 2025;279:117410. https://doi.org/10.1016/j.bios.2025.117410

2. Li X, Li Y, Wang C et al. Structure-switchable dsDNA promoter regulates the activity of CRISPR-Cas12a for APE1 detection. Talanta. 2025;294:128161. https://doi.org/10.1016/j.talanta.2025.128161

3. Shan J, Sheng Y, Luo L et al. One-pot Rlock-mediated CRISPR/Cas12a-driven RCA cycle for rapid and high-sensitive APE1 detection. Anal Chem. 2025;97:18208–18216. https://doi.org/10.1021/acs.analchem.5c03234

4. Ding S, Li H, Li J et al. One-pot detection of biomarker apurinic/apyrimidinic endonuclease 1 based on the modified-crRNA-regulated trans-cleavage activity of CRISPR/Cas12a. ACS Synth Biol. 2025;14:3186–3195. https://doi.org/10.1021/acssynbio.5c00335

5. Li X, Wang J, Cheng X et al. A multi-functional synergistic platform of Cas12a split dsDNA activators. Chem Commun. 2025;61:6615–6618. https://doi.org/10.1039/D5CC01613D
